# Synergistic Zundel–Eigen mechanism for proton transport in protic ionic liquids unraveled by machine learning force fields and NMR spectra

**DOI:** 10.1093/nsr/nwag375

**Published:** 2026-06-17

**Authors:** Junfeng Lu, Jinliang Zhang, Yumiao Lu, Jingjian Li, Yanlei Wang, Suojiang Zhang, Hongyan He

**Affiliations:** Beijing Key Laboratory of Solid State Battery and Energy Storage Process, State Key Laboratory of Mesoscience and Process Engineering, Institute of Process Engineering, Chinese Academy of Sciences, Beijing 100190, China; School of Chemical Engineering, University of Chinese Academy of Sciences, Beijing 100049, China; Beijing Key Laboratory of Solid State Battery and Energy Storage Process, State Key Laboratory of Mesoscience and Process Engineering, Institute of Process Engineering, Chinese Academy of Sciences, Beijing 100190, China; Beijing Key Laboratory of Solid State Battery and Energy Storage Process, State Key Laboratory of Mesoscience and Process Engineering, Institute of Process Engineering, Chinese Academy of Sciences, Beijing 100190, China; Department of Applied Biology and Chemical Technology, The Hong Kong Polytechnic University, Hong Kong 999077, China; School of Chemistry and Life Resources, Renmin University of China, Beijing 100872, China; Beijing Key Laboratory of Solid State Battery and Energy Storage Process, State Key Laboratory of Mesoscience and Process Engineering, Institute of Process Engineering, Chinese Academy of Sciences, Beijing 100190, China; Beijing Key Laboratory of Solid State Battery and Energy Storage Process, State Key Laboratory of Mesoscience and Process Engineering, Institute of Process Engineering, Chinese Academy of Sciences, Beijing 100190, China; School of Chemical Engineering, University of Chinese Academy of Sciences, Beijing 100049, China

**Keywords:** proton transfer mechanism, protic ionic liquids, machine-learning potential, molecular simulation, nuclear quantum effects

## Abstract

Elucidating proton transfer in nonaqueous media is essential for the rational design of advanced chemical processes and energy materials, yet its molecular mechanism remains poorly understood. Here, we combine machine-learning potential-based molecular dynamics, path-integral molecular dynamics simulations and metadynamics, and ¹H NMR spectroscopy to investigate proton-transfer dynamics in choline-amino acid protic ionic liquids. We show that proton migration occurs across a continuum of transient solvation motifs, including Eigen-like, Zundel-like, and intermediate configurations, with intermediate states being predominant. In contrast to aqueous systems, where Eigen and Zundel motifs are often comparably populated, these ionic liquids exhibit a markedly higher population of Zundel-like proton-sharing dimers than Eigen-like monomers, revealing a unique excess-proton solvation landscape in nonaqueous ionic environments. Free-energy analyses establish that molecular flexibility and steric hindrance cooperatively govern proton-transfer barriers, thereby explaining the pronounced structure–property relationships across different amino acid anions. Elevated temperature promotes proton exchange by accelerating hydrogen-bond-network reorganization, whereas nuclear quantum effects reduce the effective free-energy barrier through proton delocalization and zero-point motion, facilitating room-temperature proton transfer. ^1^H NMR measurements provide qualitative experimental evidence for rapid exchangeable-proton dynamics, consistent with the simulated local proton-exchange trends. Together, these results establish a molecular framework linking local proton solvation, ionic liquid architecture, and proton-exchange dynamics, extending the concept of Grotthuss-like proton relay beyond water and offering design principles for high-performance proton-conducting ionic liquids.

## Teaser

Machine-learning-driven simulations and NMR spectra reveal that nonaqueous proton transfer in choline–amino acid ionic liquids hinges on Zundel-like clusters modulated by flexibility, temperature, and nuclear quantum effects.

## INTRODUCTION

Proton transfer is a fundamental process in chemical and biological systems [1–8], underlying enzyme catalysis [9], acid-base equilibria [10,11], and proton conduction in energy-related and green chemistry applications [12]. In aqueous media, proton transport is commonly described by the Grotthuss mechanism [13], which accounts for the anomalously high mobility of hydrated protons [14,15]. The excess proton in water is often represented by two limiting solvation motifs: the Eigen cation, H_9_O_4_^+^ [16], in which a central H_3_O^+^ is stabilized by three hydrogen-bonded water molecules, and the Zundel cation, H_5_O_2_^+^ [17], in which a proton is nearly symmetrically shared between two water molecules. Extensive theoretical and spectroscopic studies have demonstrated that proton migration in liquid water involves rapid interconversion between Eigen and Zundel motifs [18–20], accompanied by transient proton delocalization and concerted rearrangements of the hydrogen-bond network [21]. By contrast, the microscopic mechanism of proton transport in nonaqueous solvents and complex ionic environments remains far less well understood.

Protic ionic liquids (PILs), formed through proton transfer from a Brønsted acid to a Brønsted base, possess intrinsic proton-conducting capabilities [22–25]. Among them, amino acid-based ionic liquids (AAILs) are particularly attractive owing to their biocompatibility and potential applications in biomedicine, pharmaceuticals, and energy-related technologies [26–30]. Proton mobility in AAILs is governed by labile protons embedded in dynamic hydrogen-bond networks, where many-body interactions can promote proton exchange even in the absence of complete proton dissociation [31,32]. In addition, the clustering of like-charged species, particularly amino acid anions, has been proposed to create locally connected hydrogen-bond environments that may facilitate proton migration [33,34]. Recently, by combining *ab initio* molecular dynamics (AIMD) with free-energy sampling, Yoon *et al*. [35]  showed that proton transfer in a serinate-based AAIL proceeds via a two-step pathway, in which formation of a zwitterionic intermediate precedes proton delivery to a neighboring carboxylate group. These findings suggest that, unlike proton transport in the relatively homogeneous hydrogen-bond network of water, proton exchange in PILs is governed by heterogeneous and highly cooperative local interactions. Nevertheless, a unified molecular framework that links proton solvation motifs, hydrogen-bond rearrangement, and proton-transfer energetics in PILs remains lacking.

From a methodological perspective, an accurate description of proton transfer remains challenging, particularly when bond breaking and formation, fluctuating charge localization, and nuclear quantum effects (NQEs) are involved [1,36,37]. Classical force fields are generally unable to capture the evolving electronic structure and quantum delocalization of protons, whereas fully quantum-mechanical AIMD, although accurate, is computationally too demanding to reach the length and time scales required for statistically robust sampling of rare proton-transfer events in viscous ionic liquids [1,38]. Machine-learning potentials (MLPs) have emerged as a powerful alternative [39–43], providing near-quantum-chemical accuracy while enabling simulations far beyond the time scales accessible to AIMD [1,20,44]. This capability provides new opportunities to resolve complex hydrogen-bond dynamics and competing proton-transfer pathways in PILs.

**Figure 1.**
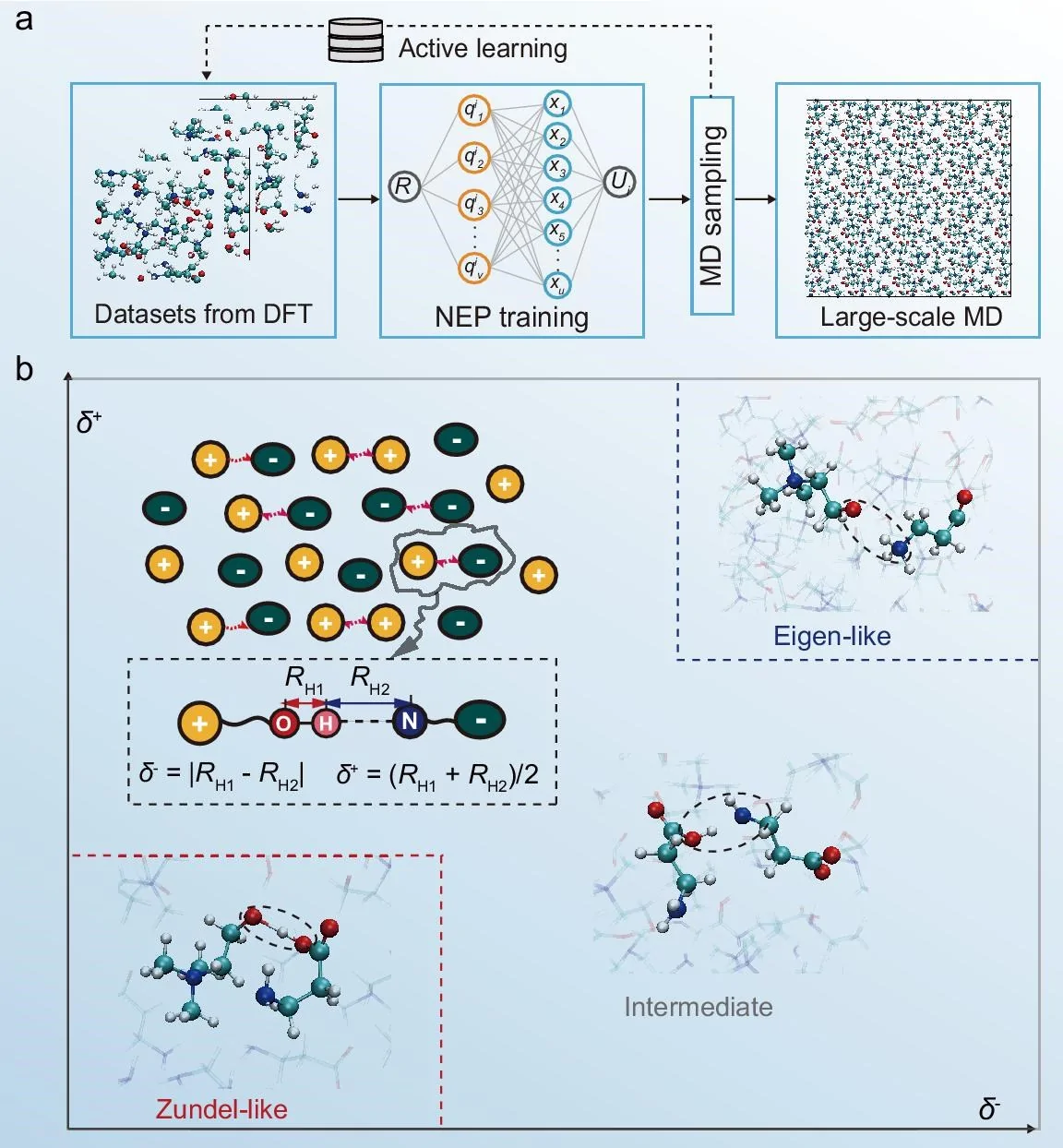
(a) Schematic workflow for the construction and validation of machine-learning potentials (MLPs), including training-set generation from density functional theory (DFT) calculations, NEP model training, and independent test-set evaluation. (b) Identification and classification of solvation structures associated with active protons during proton transfer events.

Here, we develop high-accuracy MLPs for four choline-amino acid ionic liquids (Ch-AAILs) within the neuroevolution potential (NEP) framework (Fig. 1a and Note S1) [41]. Analysis of proton trajectories reveals two distinct local transfer regimes: one dominated by reversible proton exchange and back-transfer within strongly coupled ionic dimers, and the other in which back-transfer is suppressed, thereby favoring more localized monomeric configurations. Using physically motivated geometric descriptors [45,46], we classify transient proton-solvation environments into Eigen-like, Zundel-like, and intermediate motifs. Free-energy profiles along relevant reaction coordinates are then used to quantify representative proton-transfer pathways, local transfer frequencies, and apparent free-energy barriers (Fig. 1b). By correlating these results with the heterogeneous hydrogen-bond networks of Ch-AAILs, we elucidate how local solvation dynamics, molecular flexibility, and steric constraints collectively shape the proton-transfer free-energy landscape. Temperature-dependent simulations show that thermal fluctuations promote hydrogen-bond-network reorganization, whereas path-integral molecular dynamic (PIMD) simulations reveal that NQEs reduce the effective free-energy penalty through proton delocalization and zero-point motion. Together, these results establish a molecular framework for proton exchange in nonaqueous ionic environments and provide design principles for tailoring proton-conducting ionic liquids.

## RESULTS

### Construction of machine-learning potentials for protic ionic liquids

We constructed NEP models for four Ch-AAIL systems: choline *β*-alaninate ([Ch][*β*-Ala]), choline glycinate ([Ch][Gly]), choline serinate ([Ch][Ser]), and choline prolinate ([Ch][Pro]) (Fig. S1). Reference datasets comprising energies, atomic forces, and virial stresses were generated from AIMD trajectories and additional density functional theory (DFT) single-point calculations [47,48]. The MLPs were developed following the workflow shown in Fig. 1, with sampling protocols designed to cover relevant intramolecular degrees of freedom, hydrogen-bond rearrangements, and proton-transfer-related configurations (Table S1 and Note S2). Validation against independent DFT test sets (Figs S2–S5) shows excellent agreement for both energies and forces, demonstrating the high predictive accuracy of the resulting NEP models.

**Figure 2.**
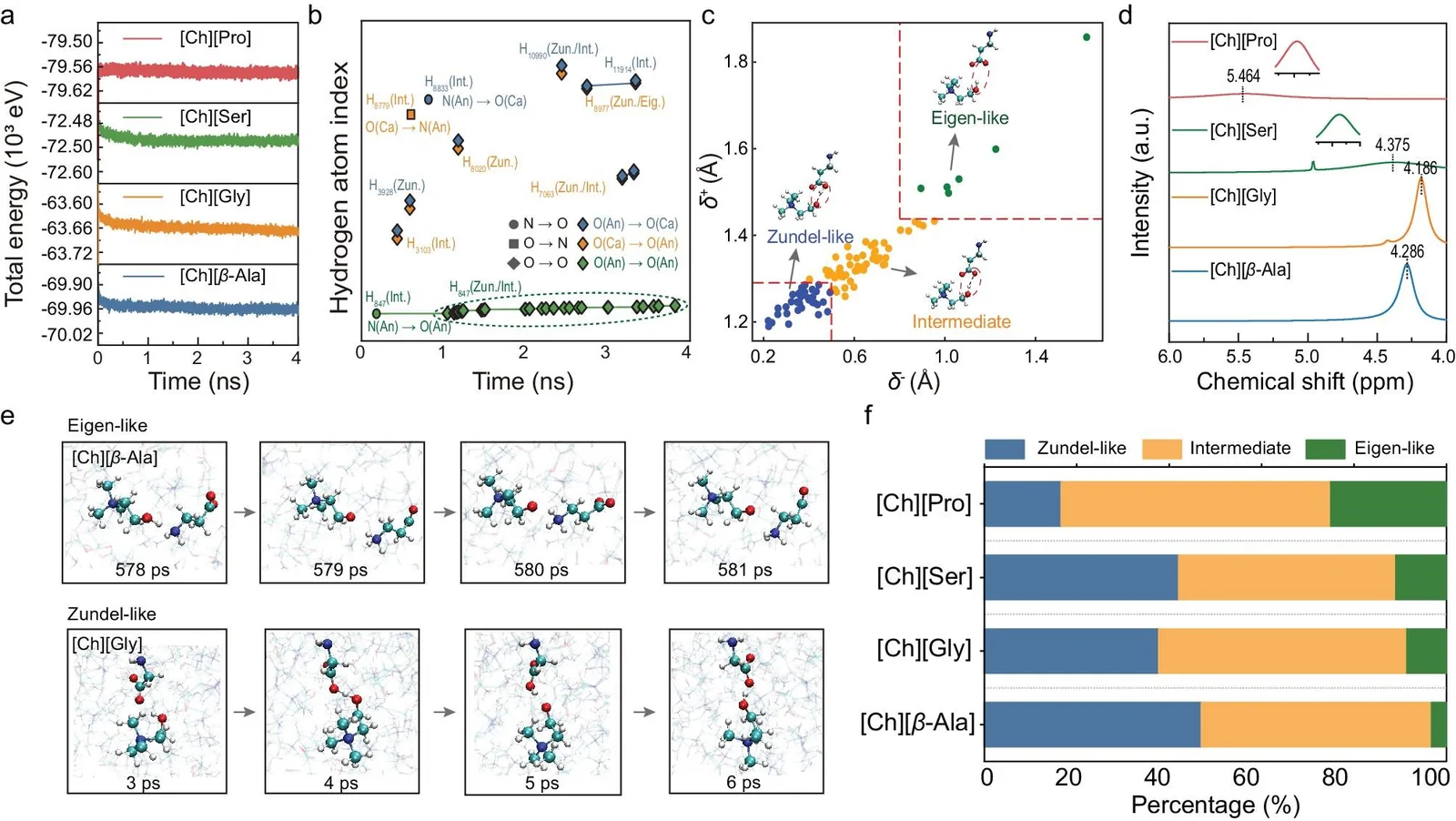
(a) Time evolution of the total energy for the four choline–amino acid ionic liquids (Ch-AAILs) during NEP-MD simulations. (b) Temporal evolution of instantaneous proton configuration types for selected active protons along a representative [Ch][*β*-Ala] trajectory. (c) Two-dimensional classification plane used to distinguish transient proton configurations. (d) Local ¹H NMR spectra (4.0–6.0 ppm region) highlighting the active proton resonance, arising from rapid exchange between the cholinium –OH group and amino acid –NH_2_ groups, for four Ch-AAILs: [Ch][*β*-Ala] (4.286 ppm), [Ch][Gly] (4.186 ppm), [Ch][Ser] (4.375 ppm), and [Ch][Pro] (5.464 ppm). (e) Time-resolved snapshots of two representative continuous proton transfer events in [Ch][*β*-Ala] (top: Eigen-like–dominated pathway; bottom: Zundel-like–dominated pathway). (f) Statistical populations of Zundel-like, intermediate, and Eigen-like configurations across the four Ch-AAILs, obtained from 4 ns trajectories.

Production NEP-based molecular dynamics simulations (NEP-MD) were subsequently performed for systems containing 384 ion pairs, which are substantially larger than the simulation cells used for AIMD sampling and therefore better suited to capturing the heterogeneous hydrogen-bond networks of the ionic liquids. Grimme’s D3 dispersion correction was included to account for dispersion interactions [49,50]. The NEP-MD trajectories remained stable in the NPT ensemble, and structural equilibration was reached within ∼1 ns for all systems (Fig. 2a), consistent with the slow relaxation dynamics characteristic of ionic liquids.

The structural fidelity of the NEP models was further evaluated by comparing radial distribution functions (RDFs) of key donor-acceptor pairs obtained from 4 ns NEP-MD trajectories with those from 10 ps AIMD simulations (Figs S6–S10). The NEP models accurately reproduce the peak positions and first-shell intensities, with minor deviations mainly attributable to the limited sampling afforded by the short AIMD trajectories [1]. Additional validation using vibrational densities of states (VDOS) (Fig. S11) and temperature-dependent densities (Fig. S12) further supports the robustness and transferability of the MLPs. The VDOS profiles provide a dynamical fingerprint of local interaction strength and hydrogen-bond-network flexibility in Ch-AAILs: low-frequency features are associated with collective intermolecular and conformational motions, whereas high-frequency features mainly arise from localized X–H vibrations modulated by hydrogen bonding. The agreement in VDOS therefore indicates that the NEP models capture not only equilibrium structural features but also local vibrational dynamics and hydrogen-bond-network fluctuations that are essential for describing the microscopic behavior of Ch-AAILs.

### Identification of proton transfer processes in protic ionic liquids

Using [Ch][*β*-Ala] as a representative system, analysis of 4 ns NEP-MD trajectories reveals frequent local proton-transfer events (Fig. S13). Two primary transfer regimes are identified: (i) proton transfer from the cationic hydroxyl group to the anionic amino group (–NH_2_), resulting in zwitterionic ion-pair formation; and (ii) proton transfer to the carbonyl oxygen of the amino acid anion, which neutralizes the amino acid anion and generates a zwitterionic choline species. These primary events further trigger secondary processes, including localized proton exchange, short-range proton-relay behavior between zwitterionic and neutral species, and intramolecular proton migration within the anion (Fig. S14).

Because proton-transfer barriers and transient proton-sharing motifs are central to this study, we further validated the NEP models in chemically reactive regions. Representative local clusters centered on proton-transfer events were extracted and recalculated at the DFT level. These clusters include configurations before, during, and after proton transfer, thereby spanning ionic, proton-shared/zwitterionic, and neutral-like local states. Direct comparisons of NEP and DFT energies and forces, together with constrained proton-transfer scans, show that the NEP models reproduce the local energetic trends and force distributions in the proton-transfer region (Figs S15–S24). These results support the reliability of the NEP models for describing local proton-sharing and proton-migration configurations.

Statistical analysis of all proton-transfer events reveals dynamic interconversion among Eigen-like, Zundel-like, and intermediate configurations (Fig. 2b). For example, proton H-847 initially transfers from the choline hydroxyl group to an –NH_2_ site, forming an intermediate zwitterionic configuration. Approximately 1 ns later, it migrates to a neighboring anion and subsequently oscillates between donor and acceptor sites for several nanoseconds, frequently sampling nearly symmetric Zundel-like geometries. In a representative Eigen-like pathway (Fig. 2e, top), the proton approaches the target –NH_2_ group at 578 ps, completes transfer by 579 ps, and becomes localized on the acceptor nitrogen. Subsequent gradual separation of the ion pair produces a monomeric Eigen-like motif with limited back-transfer. By contrast, the Zundel-like pathway (Fig. 2e, bottom) emerges when a zwitterionic anion approaches a neutral species. The donor–acceptor distance decreases rapidly within 3 ps, followed by proton hopping at 4 ps. The original anion remains strongly coupled to the transferred proton through hydrogen bonding, forming a Zundel-like dimer. Multiple oscillations and short-lived back-transfers then occur over the next 2 ps, highlighting the highly fluxional nature of the shared-proton configuration.

Transient proton configurations were quantitatively characterized using an adaptive two-dimensional (2D) geometric classification scheme inspired by approaches originally developed for aqueous proton solvation [51,52]. Hydrogen-bonded structures involving the transferring proton were classified as Zundel-like, intermediate, or Eigen-like based on two descriptors: the asymmetry coordinate, *δ*⁻ = |*R*_H1_ − *R*_H2_|, and the average bond-length coordinate, *δ*⁺ = (*R*_H1_ + *R*_H2_)/2, where *R*_H1_ and *R*_H2_ denote the distances from the transferring proton to the two donor/acceptor heavy atoms involved in the local proton-sharing unit (Fig. 2c and Fig. S25).

For each Ch-AAIL system, the *δ*⁻ and *δ*⁺ distributions were independently fitted using Gaussian mixture models (GMMs), and the intersections between adjacent Gaussian components were used as adaptive thresholds. In the resulting 2D classification scheme, configurations with low *δ*^−^ and low *δ*^+^ were assigned as Zundel-like, corresponding to nearly symmetric proton sharing within a compact donor-H-acceptor complex. Configurations with high *δ*^−^ and high *δ*^+^ were assigned as Eigen-like, reflecting a localized proton and a more weakly coupled donor-acceptor pair. The remaining configurations were classified as intermediate, representing the continuous transition region between these limiting motifs. This GMM-assisted classification provides a physically interpretable and reproducible framework for comparing transient proton-solvation environments across the four Ch-AAILs, with threshold uncertainties reported in Table S2.

Population analysis across the four Ch-AAILs (Fig. 2f) reveals that intermediate configurations are generally predominant, followed by Zundel-like and Eigen-like motifs. This distribution indicates that proton transfer in these ionic liquids is dominated by transient intermediate and proton-sharing configurations rather than by long-lived Eigen-like species. The trend differs from that typically observed in bulk water, where Eigen-like and Zundel-like motifs are often more comparably populated [19]. The substantial population of Zundel-like motifs in most Ch-AAILs reflects the heterogeneous local charge environment, asymmetric hydrogen-bond networks, and steric constraints imposed by amino acid side chains, which together favor local proton sharing and back-transfer. Notably, [Ch][Pro] exhibits a distinct profile, with a reduced Zundel-like population and a higher Eigen-like fraction. This behavior is attributed to the lower proton affinity of its acceptor sites and the conformational rigidity of the proline ring, which suppress productive donor–acceptor reorganization and destabilize symmetric proton-sharing motifs.

To provide experimental support for the rapid exchangeable-proton dynamics suggested by NEP-MD simulations, we performed ^1^H NMR measurements (Fig. 2d and Fig. S26). The exchangeable protons associated with the choline –OH group and amino acid –NH_2_ groups coalesce into a single averaged resonance, indicating rapid intermolecular exchange on the NMR timescale. This behavior is consistent with fast exchange commonly observed for labile protons in hydrogen-bonded systems [53]. Among the four systems, [Ch][Gly] exhibits the sharpest and most intense resonance, suggesting faster exchangeable-proton dynamics on the NMR timescale. [Ch][*β*-Ala] shows a moderately attenuated signal, whereas [Ch][Ser] and [Ch][Pro] display more pronounced line broadening, consistent with slower exchange dynamics. These experimental trends provide qualitative support for the relative proton-exchange activities predicted by the NEP-MD simulations, although the NMR spectra do not directly resolve individual transient Eigen-like, Zundel-like, or intermediate configurations.

### Microscopic mechanism of proton transfer in protic ionic liquids

**Figure 3.**
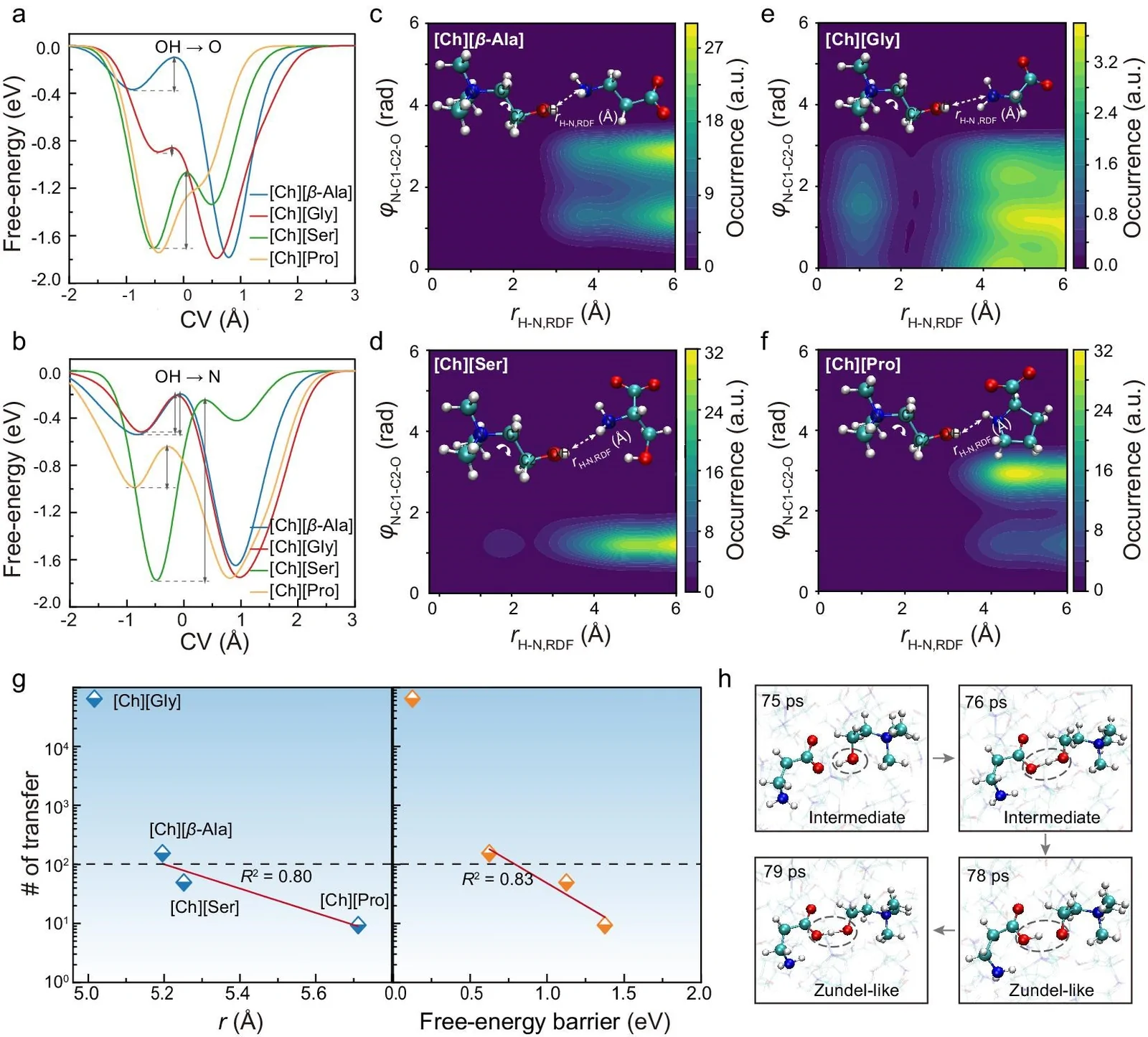
(a, b) Free-energy profiles for the two dominant proton-transfer pathways: (a) transfer from the cationic hydroxyl group to the anionic carbonyl oxygen (OH ↔ O) and (b) transfer from the cationic hydroxyl group to the anionic amino group (OH ↔ NH_2_). (c–f) Two-dimensional combined distribution functions characterizing the pre-coordination stage, plotted as a function of the donor–proton–acceptor nitrogen distance (*r*_H–N_) and the key cationic dihedral angle *φ*_N–C1–C2–O_ (in radians). (g) Quantitative correlation among proton transfer frequency, the position of the first cation–anion RDF peak, and the corresponding free-energy barrier. (h) A representative continuous proton transfer event in [Ch][*β*-Ala] obtained under enhanced sampling conditions.

Proton-transfer efficiency is governed by the coupled effects of transfer energetics, local hydrogen-bond preorganization, and steric constraints. To map representative free-energy landscapes, we performed PLUMED-enhanced metadynamics simulations (Fig. 3a and b) [54]. For the hydroxyl-to-carbonyl oxygen pathway (OH ↔ O), the effective free-energy barriers for [Ch][*β*-Ala], [Ch][Gly], [Ch][Ser], and [Ch][Pro] are 0.49, 0.04, 0.63, and 1.78 eV, respectively. The exceptionally low barrier in [Ch][Gly] is consistent with its higher electrical conductivity [55], whereas the large barrier in [Ch][Pro] indicates strong suppression of this channel. For the hydroxyl-to-amino pathway (OH ↔ NH_2_), the corresponding barriers are 0.34, 0.31, 1.53, and 0.34 eV, respectively. In this pathway, [Ch][Gly] again exhibits a low barrier, whereas [Ch][Ser] shows a substantially larger barrier, indicating a strongly hindered OH ↔ NH_2_ transfer pathway. These values are interpreted as effective condensed-phase free-energy barriers along selected collective variables, incorporating contributions from proton displacement, local hydrogen-bond-network reorganization, steric constraints, and entropic effects. They are therefore used primarily to compare the relative accessibility of different proton-transfer channels rather than as direct one-to-one predictors of absolute transfer rates.

To elucidate how intramolecular conformational flexibility modulates proton transfer, we constructed two-dimensional combined distribution functions (2D-CDFs) [29] correlating the proton–acceptor nitrogen distance, *r*_H–N_, with the key cationic dihedral angle, *φ*_N–C1–C2–O_ (Fig. 3c–f). The former reflects instantaneous H…N pre-coordination, whereas the latter describes the rotational flexibility of the choline headgroup. A clear correlation emerges: broader dihedral distributions facilitate the formation of short H…N contacts, thereby generating productive precursor geometries for proton transfer. In contrast, restricted conformational sampling produces narrower and more disconnected high-density regions in the 2D-CDFs, indicating steric obstruction of productive donor–acceptor arrangements. The flexibility trend, [Ch][Gly] > [Ch][*β*-Ala] > [Ch][Ser] > [Ch][Pro], follows the inverse steric sequence and is consistent with the H–N coordination strength (Fig. S27) and the OH ↔ NH_2_ free-energy barriers. These results indicate that conformational flexibility promotes transient pre-coordination and lowers the effective free-energy cost of proton transfer.

For the OH ↔ O channel, analogous 2D-CDF and RDF analyses based on *r*_H–O_ (Figs S28 and S29) yield the flexibility sequence [Ch][Gly] > [Ch][Pro] > [Ch][*β*-Ala] > [Ch][Ser], consistent with the corresponding first-shell peak intensities. Notably, [Ch][Pro] exhibits relatively strong H…O coordination at ∼1.6 Å despite its large OH ↔ O free-energy barrier. This apparent discrepancy can be rationalized by the rigidity of the five-membered pyrrolidine ring. Although the carbonyl oxygen can form stable hydrogen bonds with choline, ring rigidity impedes the geometric reorganization required to access productive transfer configurations. Thus, strong local coordination in [Ch][Pro] can lead to hydrogen-bond trapping rather than efficient proton migration. This observation highlights that steric effects are not merely static distance constraints but also dynamically control the rearrangements required for proton transfer.

The relationship among local proton-transfer frequency, the position of the first cation–anion RDF peak, and the corresponding free-energy barrier is summarized in Fig. 3g and Fig. S30. For the OH ↔ O channel, excluding [Ch][Gly], whose ultralow barrier makes it an outlier, the remaining systems show that shorter characteristic cation–anion coordination distances are generally associated with lower free-energy barriers and higher local transfer counts. This trend suggests that short-range hydrogen-bond preorganization facilitates proton exchange, although local transfer frequency should not be regarded as a direct measure of macroscopic proton conductivity.

To relate the simulated proton-transfer activity to experimental transport behavior, we plotted the number of local proton-transfer events against the measured ionic conductivities of the four Ch-AAILs (Fig. S31) [55]. The observed positive qualitative correlation suggests that systems with more frequent local proton exchange tend to exhibit higher ionic conductivity. However, this comparison should not be interpreted as a direct quantitative prediction of macroscopic proton transport, because local transfer counts do not explicitly distinguish persistent forward transfer, back-transfer, local oscillation, or net long-range charge displacement.

A representative transfer event in [Ch][*β*-Ala] (Fig. 3h) illustrates the local interconversion sequence Eigen-like ↔ intermediate ↔ Zundel-like. Starting from a localized Eigen-like configuration, the proton evolves through an intermediate state and transiently forms a nearly symmetric Zundel-like dimer as the donor-acceptor distance decreases. This trajectory reveals a localized proton-sharing and short-range relay process in Ch-AAILs, highlighting the role of Zundel-like dimers as transient intermediates that facilitate local proton migration.

### Temperature and nuclear quantum effects on proton transfer

**Figure 4.**
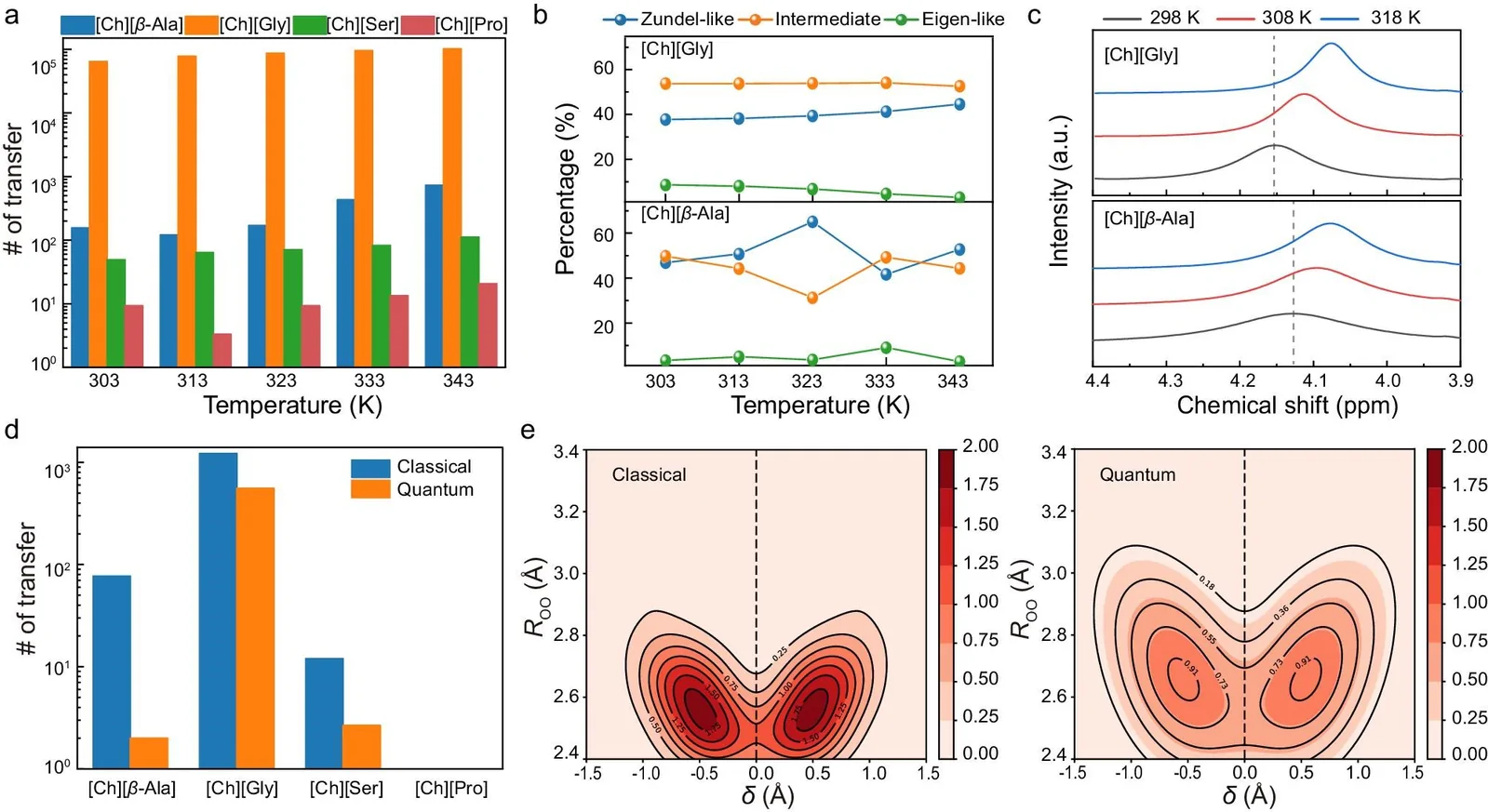
(a) Temperature dependence of the total number of proton transfer events. (b) Temperature-dependent populations of Zundel-like, intermediate, and Eigen-like configurations in [Ch][Gly] and [Ch][*β*-Ala]. (c) Variable-temperature ¹H NMR spectra of [Ch][Gly] (top) and [Ch][β-Ala] (bottom), recorded in DMSO-*d*_6_ over the 3.9–4.4 ppm region at 298, 308, and 318 K, shown from bottom to top. (d) Comparison of local proton-transfer counts obtained from classical and path-integral NEP-MD simulations. (e) Free-energy landscapes along the O–H…O proton-transfer coordinate and O…O separation in [Ch][*β*-Ala] obtained from classical and path-integral NEP-MD simulations; color bars indicate relative free energy in eV.

The influence of temperature on proton-transfer dynamics was examined using NEP-MD simulations over the range of 303 to 343 K. As shown in Fig. 4a, the number of local proton-transfer events increases markedly with temperature in all systems. Representative proton trajectories in [Ch][*β*-Ala] (Fig. S32) further show that elevated temperature enhances thermal fluctuations and accelerates hydrogen-bond-network reorganization, thereby increasing the probability of successful interionic proton exchange.

Temperature-dependent populations of the three transient proton-solvation motifs are summarized in Fig. 4b and Fig. S33. In [Ch][Gly], which exhibits the highest overall transfer activity, intermediate configurations remain dominant across the entire temperature range, accounting for ∼50% of the sampled proton-transfer configurations. The hierarchy of intermediate > Zundel-like >> Eigen-like is preserved in [Ch][Gly] with Eigen-like fractions remaining below 10% at all temperatures. By contrast, [Ch][*β*-Ala] and [Ch][Ser] show stronger temperature sensitivity, with Zundel-like populations becoming comparable to, or even exceeding, intermediate populations at elevated temperatures. [Ch][Pro] also shows temperature-dependent variations, but with larger numerical fluctuations because of its limited event statistics and the sensitivity of the two-dimensional classification scheme to small sample sizes. These variations are therefore interpreted as quantitative fluctuations rather than clear evidence of a mechanistic transition.

As shown in Fig. 4c and Fig. S34, variable-temperature ^1^H NMR measurements provide qualitative experimental support for enhanced exchangeable-proton dynamics at elevated temperatures. With increasing temperature, the coalesced exchangeable-proton resonance undergoes a high-field shift, accompanied by line broadening and increased signal intensity. These changes are consistent with accelerated exchange of labile protons on the NMR timescale, as reported for other hydrogen-bonded systems [56]. Because NMR does not directly resolve individual transient proton-sharing motifs under fast-exchange conditions, these spectra are used here as qualitative support for enhanced proton exchange rather than as direct structural evidence for the simulated Eigen-like, Zundel-like, or intermediate populations.

The role of NQEs was further evaluated using PIMD simulations. We performed 200 ps PIMD simulations at 303 K and compared them with classical trajectories at the same temperature (Fig. 4d). Inclusion of quantum nuclear motion increases the local proton-transfer frequency in [Ch][Gly], [Ch][Ser], and [Ch][*β*-Ala]. In contrast, no proton-transfer events were observed for [Ch][Pro] within the 200 ps PIMD trajectory, consistent with its large classical barrier and strong steric constraints. This absence indicates kinetic suppression on the simulated timescale rather than the absence of a possible quantum-assisted pathway.

Following the approach of Marx *et al*. [51], we further analyzed the joint probability distribution using *δ* = *R*_OaH_ − *R*_ObH_ as the proton-transfer coordinate and *R*_OO_ as the donor-acceptor separation. Here, *δ* describes the displacement of the shared proton relative to the two oxygen atoms, whereas *R*_OO_ denotes the Oa…Ob distance between the donor and acceptor oxygen atoms. For [Ch][*β*-Ala] (Fig. 4e), the effective free-energy barrier decreases from ∼1.50 eV in classical MD to ∼0.91 eV in PIMD. This reduction should be interpreted as an effective lowering of the free-energy barrier induced by nuclear quantum fluctuations, including proton delocalization and zero-point motion, rather than as a change in the underlying Born–Oppenheimer potential energy surface. Proton delocalization and zero-point motion allow the shared proton to sample transition-region configurations more efficiently, thereby facilitating proton transfer.

These results demonstrate that NQEs play an important role in lowering the effective free-energy penalty for proton transfer in Ch-AAILs. We note, however, that PIMD provides an equilibrium sampling description of quantum nuclear fluctuations and does not, by itself, constitute direct dynamical evidence for tunneling-controlled kinetics. Therefore, the present results support NQE-assisted proton transfer, with possible tunneling contributions, rather than establishing a strictly tunneling-dominated mechanism.

## CONCLUSIONS

By integrating high-fidelity MLPs, enhanced sampling, path-integral simulations, and ¹H NMR spectroscopy, this study elucidates the microscopic proton-transfer dynamics in nonaqueous PILs. A central finding is the overall hierarchy of transient proton-solvation motifs—intermediate > Zundel-like > Eigen-like—which indicates that proton exchange in Ch-AAILs is dominated by transient intermediate and proton-sharing configurations rather than by long-lived Eigen-like species. This behavior contrasts with the more balanced Eigen–Zundel interplay commonly discussed for aqueous proton solvation and reveals a distinct excess-proton solvation landscape in heterogeneous ionic environments.

Our results further show that proton-transfer barriers are governed not by local bonding alone but by the cooperative interplay of hydrogen-bond preorganization, molecular flexibility, and steric constraints. Flexible ionic architectures promote productive donor-acceptor configurations and lower the effective free-energy cost of proton transfer, whereas steric rigidity can trap hydrogen-bonded structures and suppress productive migration. Temperature enhances local proton exchange by promoting hydrogen-bond-network reorganization, whereas PIMD simulations reveal that NQEs substantially reduce the effective free-energy penalty through proton delocalization and zero-point motion. These results support an NQE-assisted proton-transfer mechanism in Ch-AAILs, without establishing strictly tunneling-controlled kinetics.

Taken together, local proton migration in Ch-AAILs proceeds through a dynamic local relay sequence—localized Eigen-like ↔ intermediate ↔ Zundel-like ↔ another localized Eigen-like state—that resembles a short-range Grotthuss-like proton-relay process but is strongly modulated by asymmetric hydrogen bonding, chemical heterogeneity, steric constraints, and quantum nuclear fluctuations. This framework rationalizes the sensitivity of proton-exchange dynamics to anion identity and molecular architecture and provides atomistic design principles for tailoring proton-conducting ionic liquids. More broadly, this work extends the molecular picture of proton transport beyond aqueous systems and offers guidance for engineering tunable proton-conducting media for green chemistry and sustainable energy applications.

## METHODS

### Machine-learning interatomic potentials

Machine-learning interatomic potentials were constructed within the NEP framework implemented in GPUMD [41]. Separate NEP models were developed for four Ch-AAILs, namely [Ch][*β*-Ala], [Ch][Gly], [Ch][Ser], and [Ch][Pro]. The models express the total potential energy as a sum of atomic site energies represented by neural networks of local many-body descriptors and were trained against DFT reference energies, atomic forces, and virial stresses.

### Training dataset generation

Initial configurations were generated using classical molecular dynamics with the OPLS-AA force field [57,58] in LAMMPS [59]. Representative structures were then sampled from AIMD trajectories performed in VASP using PAW pseudopotentials, the PBE exchange–correlation functional, and DFT-D3 dispersion corrections [48,50,60]. The D3-corrected AIMD simulations were used to generate physically reasonable liquid configurations and hydrogen-bonded local environments. To improve configurational coverage, AIMD sampling was conducted over 300–800 K, and selected snapshots were augmented by lattice strains and small random atomic displacements. High-precision single-point DFT calculations were subsequently performed using the PBE functional without D3 corrections to generate the reference labels for NEP training and validation. Dispersion interactions were therefore not included in the NEP training labels but were added explicitly during subsequent NEP-MD simulations to avoid double counting.

### Model training and molecular dynamics simulations

NEP models were trained using the separable natural evolution strategy [61], and their robustness was improved through active learning involving iterative NEP-MD sampling, DFT relabeling, and retraining. Production NEP-MD simulations were performed in GPUMD for systems containing 384 ion pairs with a 0.5 fs time step with explicit DFT-D3 corrections included. Each system was equilibrated in the NPT ensemble and subsequently simulated in the NVT ensemble for structural and dynamical analyses. NQEs were evaluated using PIMD simulations with i-PI interfaced to GPUMD [62], representing each atom by a 24-bead ring polymer.

### Free-energy calculations

Proton-transfer free-energy profiles were computed using well-tempered metadynamics as implemented in PLUMED2 [54]. The simulations employed the converged NEP models with explicit DFT-D3 corrections. Collective variables were defined as differences between donor-proton and proton-acceptor distances, and initial pathways were explored using moving restraints before production metadynamics simulations.

### Materials and synthesis

Choline hydroxide solution (44 wt%), *β*-alanine, glycine, DL-serine, and D-proline were purchased from commercial suppliers and used without further purification. Choline–amino acid ionic liquids were synthesized by acid–base neutralization through the slow addition of choline hydroxide to aqueous amino acid solutions under ice-bath conditions. The products were purified by solvent precipitation and dried under vacuum over phosphorus pentoxide.

### Nuclear magnetic resonance spectroscopy

¹H NMR spectra were recorded on a Bruker AVANCE III HD 600 MHz spectrometer. Samples were prepared by dissolving the ionic liquids in DMSO-*d*_6_ at a concentration of 8 mmol mL^−1^ and the resulting solutions were transferred to 5 mm NMR tubes. Variable-temperature measurements were performed where indicated.

## Supplementary Material

nwag375_Supplemental_File
