# Supplementary material for: Synergistic Zundel–Eigen mechanism for proton transport in protic ionic liquids unraveled by machine learning force fields and NMR spectra
Source: Natl Sci Rev. 2026 Jun 17;13(17):nwag375. doi: 10.1093/nsr/nwag375 (PMC13573309; doi:10.1093/nsr/nwag375)
Supplement: Supplementary file 1 [file nwag375_supplemental_file.pdf]

Supporting Information for

Synergistic Zundel–Eigen Mechanism for Proton Transport in Protic Ionic Liquids Unraveled by Machine Learning Force Fields and NMR Spectra

Junfeng Lu[1,2], Jinliang Zhang[1], Yumiao Lu[1], Jingjian Li[3], Yanlei Wang[,4], Suojiang Zhang[*,1] and Hongyan He[*,1,2]*

[1]Beijing Key Laboratory of Solid State Battery and Energy Storage Process, State Key Laboratory of Mesoscience and Process Engineering, Institute of Process Engineering, Chinese Academy of Sciences, Beijing 100190, China

[2] School of Chemical Engineering, University of Chinese Academy of Sciences, Beijing 100049, China

[3]Department of Applied Biology and Chemical Technology, The Hong Kong Polytechnic University, Hong Kong 999077, China

[4]School of Chemistry and Life Resources, Renmin University of China, Beijing, 100872, China

***Corresponding authors**. Email: hyhe@ipe.ac.cn (H. H.), sjzhang@ipe.ac.cn (S. Z.) and ylwang17@ruc.edu.cn (Y. W.)

Supporting Notes

Note S1. Theoretical Framework of the Neuroevolution Potential

The neuroevolution potential (NEP), first introduced by Fan and co-workers in 2021 [1] and subsequently refined in later developments [2-4], is a machine-learned interatomic potential that combines artificial neural networks with physically motivated many-body descriptors. This framework enables near-first-principles accuracy while retaining the computational efficiency required for large-scale molecular dynamics simulations.

Similar to empirical many-body potentials, NEP expresses the total potential energy, U, as a sum of atomic site energies:

$$U_{\text{total}} = \sum_{\text{i}} U_{\text{i}} \qquad \text{(S1)}$$

where U_i denotes the site energy of atom i, which depends on its local chemical environment. In NEP, the site energy is represented as a nonlinear function of local structural descriptors:

$$U_{\text{i}} = f(q_{\text{i}}) \qquad \text{(S2)}$$

where q_i is the descriptor vector characterizing the local atomic environment around atom i. The nonlinear mapping f is realized by a feed-forward neural network. For a single-hidden-layer network with $n$neurons, the site energy can be written as:

$$U_{\text{i}} = \sum_{u=1}^{n} w_u^{(1)} \tanh\left(\sum_{V} w_{uv}^{(0)} q_{\text{iv}} + b_u^{(0)}\right) + b^{(1)} \qquad \text{(S3)}$$

where $\tanh(x)$ is the activation function, $w^{(0)}$ and $w^{(1)}$ are the weight parameters connecting the input, hidden, and output layers, and $b^{(0)}$and $b^{(1)}$are the corresponding bias terms. The network parameters are optimized to minimize deviations between NEP predictions and first-principles reference data, including total energies, atomic forces, and virial stresses. This formulation allows NEP to describe complex potential-energy surfaces involving hydrogen bonding, molecular flexibility, and proton-transfer-related configurations.

Note S2. Workflow for NEP Model Development

NEP models were developed for four representative choline–amino acid ionic liquids (Ch-AAILs): choline β-alaninate ([Ch][β-Ala]), choline glycinate ([Ch][Gly]), choline serinate ([Ch][Ser]), and choline prolinate ([Ch][Pro]). All models were trained using the GPUMD package and its integrated NEP module [2]. The molecular structures of the four Ch-AAILs are shown in **Fig. S1**.

The overall workflow for NEP model development is illustrated in **Fig. 1** of the main text and consists of the following steps:

Initial configuration sampling.

Equilibrated liquid configurations were first generated using classical molecular dynamics simulations with the OPLS-AA force field. These configurations served as the starting structures for subsequent first-principles sampling.

AIMD sampling and representative frame extraction.

Ab initio molecular dynamics simulations were performed using the Vienna Ab initio Simulation Package (VASP) [5, 6]. To enhance configurational diversity and reduce extrapolation during NEP-MD simulations, AIMD sampling was conducted over a temperature range of 300–800 K. The elevated-temperature trajectories were used as an accelerated sampling strategy to include distorted hydrogen-bond networks, high-energy conformations, and proton-transfer-relevant local geometries, rather than to represent experimental operating conditions.

Dataset construction.

Representative configurations were extracted from the AIMD trajectories and further augmented by applying ±5% lattice strains and random atomic displacements of 0.03 Å. High-accuracy single-point DFT calculations were then performed for both unperturbed and perturbed structures to generate reference energies, atomic forces, and virial stresses. The resulting dataset was divided into training and validation subsets.

Model training and active learning.

Initial NEP models were trained in the GPUMD package. To improve robustness and transferability, an active-learning workflow was employed. Preliminary NEP models were used to perform MD sampling, after which structurally diverse configurations were selected using farthest-point sampling. These selected configurations were recalculated at the DFT level and incorporated into the training set after removing abnormal geometries or poorly converged structures with NeptrainKit [7].

Convergence and validation.

The simulation–sampling–labeling–retraining cycle was repeated until the prediction errors for energies, forces, and virial stresses on independent validation sets reached convergence. The final NEP models were then used for large-scale production simulations. Their reliability was further assessed against independent DFT test sets, radial distribution functions, vibrational densities of states, temperature-dependent densities, and representative local proton-transfer clusters.

Note S3. Training Dataset Preparation

High-quality and sufficiently diverse training datasets are essential for developing accurate

and transferable NEP models.

(1) Classical pre-sampling.

Initial liquid configurations were generated using LAMMPS [8] with the all-atom nonpolarizable OPLS-AA force field [9, 10]. Simulations were performed at 300 K and 1 atm. van der Waals interactions were described by 12–6 Lennard-Jones potentials with a 1.2 nm cutoff, and long-range electrostatics were treated using the particle–particle particle–mesh (PPPM) method. Each system was first relaxed by conjugate-gradient energy minimization, followed by 4 ns of NPT equilibration and 4 ns of NVT production sampling. Representative configurations from these trajectories were used as starting structures for subsequent AIMD simulations.

(2) DFT sampling and dataset augmentation.

AIMD simulations were carried out in VASP using projector-augmented-wave (PAW) pseudopotentials [11] and the Perdew–Burke–Ernzerhof (PBE) exchange–correlation functional [12], with DFT-D3 dispersion corrections included during the AIMD sampling stage [13]. The use of D3-corrected AIMD was intended to generate physically reasonable liquid configurations and hydrogen-bonded local environments for subsequent dataset construction. A plane-wave cutoff of 500 eV and Γ-point sampling were used for AIMD. The electronic convergence threshold was set to 0.01 meV atom^{-1}.

To enhance configurational diversity, NVT AIMD simulations were performed over 300–800 K, yielding approximately 800 snapshots extracted at 100 fs intervals. The elevated-temperature sampling was used to include high-energy and proton-transfer-relevant local geometries and to reduce extrapolation in subsequent NEP-MD simulations. In addition, 100 representative frames from the 300 K trajectory were augmented by applying ±5% lattice strains and random atomic displacements of 0.03 Å, yielding approximately 700 configurations in total.

High-precision single-point DFT calculations were then performed for both original and perturbed structures using the PBE functional without D3 dispersion corrections. A 3 × 3 × 3 Monkhorst–Pack k-point mesh and a tighter electronic convergence threshold of 0.001 meV atom^{-1} were used for these single-point calculations. The resulting PBE energies, atomic forces, and virial stresses were used as reference labels for NEP training and validation. Therefore, dispersion interactions were not included in the NEP training labels but were instead added explicitly during subsequent NEP-MD simulations, avoiding double counting of dispersion contributions.

Note S4. NEP Model Training

NEP training was performed in GPUMD using the separable natural evolution strategy

(SNES) optimizer [14]. Radial and angular cutoffs were set to 6.0 and 4.0 Å, respectively. Three-, four-, and five-body angular descriptors were included to capture many-body interactions, with the maximum angular momenta set to $l_{max}^{3b} = 4l_m$, $l_{max}^{4b} = 2$ and $l_{max}^{5b} = 1$.

The neural network consisted of a single hidden layer with 30 neurons. Short-range repulsive interactions were regularized using spline interpolation with the Ziegler–Biersack–Littmark (ZBL) universal potential [15]. The dataset was randomly divided into training and test subsets at a 9:1 ratio. The loss function was constructed to minimize deviations in energies, atomic forces, and virial stresses, and the test-set errors were monitored throughout training to avoid overfitting.

The converged NEP models provide high accuracy and transferability for describing the structural and dynamical properties of the studied ionic liquids. The complete set of hyperparameters is summarized in **Table S1**.

Note S5. Iterative Optimization via Active Learning

Model robustness was further improved through an active-learning workflow implemented in GPUMD, consisting of iterative cycles of model training, configuration sampling, outlier rejection, DFT labeling, and retraining. Initial validation was performed using 10 ps NPT simulations of 6-ion-pair systems at 300 K and 1 atm, with D3 dispersion corrections included [13, 16]. Structurally diverse configurations were selected by farthest-point sampling and recalculated at the DFT level.

Configurations with abnormal geometries, poor DFT convergence, or excessively large energy or force deviations were removed using NeptrainKit [7]. The remaining validated structures were added to the original training set for subsequent retraining. As the model accuracy improved, the NEP-MD sampling duration was gradually extended from short 10 ps tests to 1 ns intermediate simulations and finally to 10 ns validation trajectories, enabling increasingly stringent assessment of long-time stability in ionic-liquid environments. The active-learning cycles were continued until the energy, force, and virial-stress errors reached convergence and the NEP-MD trajectories remained stable over extended simulations. The resulting NEP models exhibit robust transferability for large-scale simulations of the four Ch-AAILs.

Note S6. NEP-MD Production Simulation Details

Large-scale production simulations were performed in GPUMD using the converged NEP models with explicit DFT-D3 dispersion corrections. Each system contained 384 ion pairs and was propagated with a time step of 0.5 fs at 303, 313, 323, and 343 K. Because the NEP models were trained on PBE single-point reference data without D3 corrections, the explicit D3 term

was added during NEP-MD simulations to account for dispersion interactions without double counting.

The simulation protocol consisted of two stages:

(1) Equilibration. Each system was equilibrated for 2 ns in the NPT ensemble using stochastic cell rescaling (SCR) barostatting [17] and Nosé–Hoover chain thermostats to achieve volume and energy equilibration.

(2) Production. The equilibrated systems were then simulated for an additional 2 ns in the NVT ensemble using Nosé–Hoover chain thermostatting. Trajectories from the NVT production stage were used for all structural, dynamical, and thermodynamic analyses.

Vibrational densities of states (VDOS) were calculated from the Fourier transform of the velocity autocorrelation function (VACF):

$$DOS(\omega) = \int_{-\infty}^{+\infty} \langle v(0)v(t) \rangle e^{-iwt} dt \qquad \text{(S4)}$$

where $\langle \mathbf{v}(0) \cdot \mathbf{v}(t) \rangle$ is the VACF. Ensemble averaging over the NVT production trajectories was used to obtain atom-resolved vibrational spectra.

Note S7. Path-Integral Molecular Dynamics (PIMD) Simulations

Nuclear quantum effects (NQEs) were incorporated using path-integral molecular dynamics (PIMD) [18], with i-PI as the driver and GPUMD as the force evaluator. Each atom was represented by a 24-bead ring polymer. Simulations were performed at 303 K and 1 bar with a time step of 0.5 fs. PIMD simulations were performed using the converged NEP models with explicit DFT-D3 dispersion corrections, consistent with the classical NEP-MD production simulations.

The PIMD workflow began with energy minimization, followed by 200,000 NPT equilibration steps and 200,000 NVT production steps, corresponding to 100 ps for each stage. During the equilibration stage, temperature and pressure were controlled using a Langevin thermostat and a Berendsen barostat, respectively. The subsequent NVT production trajectories were used for analysis. A 24-bead ring-polymer representation was used to capture the quantum nuclear fluctuations relevant to the energetic and structural distributions of the present ionic-liquid systems.

Note S8. Enhanced Sampling with PLUMED

Free-energy profiles for proton transfer were computed using PLUMED2 [19] integrated with the i-PI/GPUMD framework. The simulations employed the converged NEP models together with explicit DFT-D3 dispersion corrections, consistent with the production NEP-MD simulations. The collective variables (CVs) were defined as distance differences describing the migration of the transferring proton, H_d, from the donor oxygen atom, O_d, to either an

acceptor oxygen atom, O_a, or an acceptor nitrogen atom, N_a:

$$\mathrm{CV_O} = d(\mathrm{H}_d - \mathrm{O}_d) - d(\mathrm{H}_d - \mathrm{O}_a) \quad \text{(S5)}$$

$$\mathrm{CV_N} = d(\mathrm{H}_d - \mathrm{O}_d) - d(\mathrm{H}_d - \mathrm{N}_a) \quad \text{(S6)}$$

With this definition, negative CV values correspond to donor-bound configurations, whereas positive CV values correspond to acceptor-bound configurations. Initial reaction pathways were first explored using the MOVINGRESTRAINT method, in which the CV was gradually biased from approximately −1.0 Å, corresponding to a donor-bound state, to approximately 1.0 Å, corresponding to an acceptor-bound state.

Well-tempered metadynamics was then performed using Gaussian hills with a height of 0.005 eV, a width of $\sigma = 0.3$Å, a bias factor of 10, and a deposition interval of 300 steps. Bias deposition was allowed over the CV range of −3 to 3 Å. The CV trajectories and bias potentials were recorded every 10 steps for subsequent free-energy reconstruction. This protocol was used to enhance sampling of representative proton-transfer pathways and to obtain the corresponding effective free-energy profiles.

Note S9. Materials and Chemicals

Choline hydroxide aqueous solution (44 wt%), β-alanine (≥99%), glycine (≥99%), DL-serine (≥98%), D-proline (≥99%), and phosphorus pentoxide (P_2O_5, ≥98%) were purchased from Shanghai Aladdin Biochemical Technology Co., Ltd. HPLC-grade acetonitrile and methanol were obtained from Beijing MREDA Technology Co., Ltd. Deionized water used throughout the experiments was prepared in the laboratory.

Note S10. Synthesis of Choline-Amino Acid Ionic Liquids

[Ch][β-Ala], [Ch][Gly], [Ch][Ser], and [Ch][Pro] were synthesized via an acid-base neutralization method. A predetermined volume of choline hydroxide aqueous solution was added dropwise to an aqueous solution of the corresponding amino acid with continuous stirring in an ice-water bath at 0-5 °C, maintaining a molar ratio of choline to amino acid of 1.00:1.01 (with a slight excess of the amino acid). After the addition was complete, the reaction mixture was stirred at the same temperature for an additional 24 h. Water was then removed by rotary evaporation at 50 °C to afford a pale-yellow crude product. To remove excess amino acid, an acetonitrile/methanol mixed solvent (v/v = 7:3) was added to the crude product, followed by magnetic stirring for 12 h to precipitate unreacted amino acid; the resulting suspension was filtered under reduced pressure, and this purification step was repeated three times. The combined filtrates were concentrated by rotary evaporation at 50 °C to remove the organic solvents, yielding a viscous yellow liquid, which was finally dried in a vacuum oven over P_2O_5 at 60 °C for 48 h to remove residual moisture.

Note S11. Nuclear Magnetic Resonance (NMR) Characterization

^{1}H NMR spectra of the ionic liquids were recorded using a Bruker AVANCE III HD 600 MHz liquid-state NMR spectrometer. NMR samples were prepared by dissolving the ionic liquids in deuterated dimethyl sulfoxide (DMSO-d_6) with an ionic liquid loading of 8 $mmol \cdot mL^{-1}$ of solvent, and the resulting solutions were transferred into standard 5 mm NMR tubes for analysis. All spectra were acquired at ambient temperature unless otherwise noted.

Supporting Figures and Captions

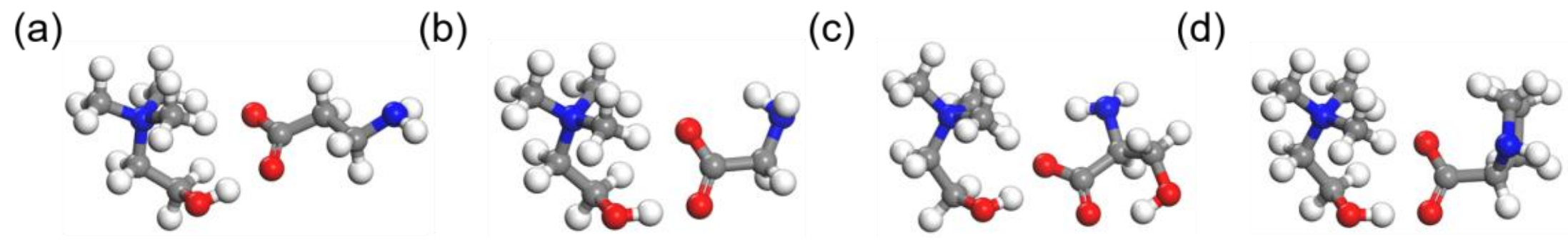

Fig. S1 Molecular structures of the four Ch-AAILs. Molecular structures of (a) choline β-alaninate ([Ch][β-Ala]), (b) choline glycinate ([Ch][Gly]), (c) choline serinate ([Ch][Ser]), and (d) choline prolinate ([Ch][Pro]).

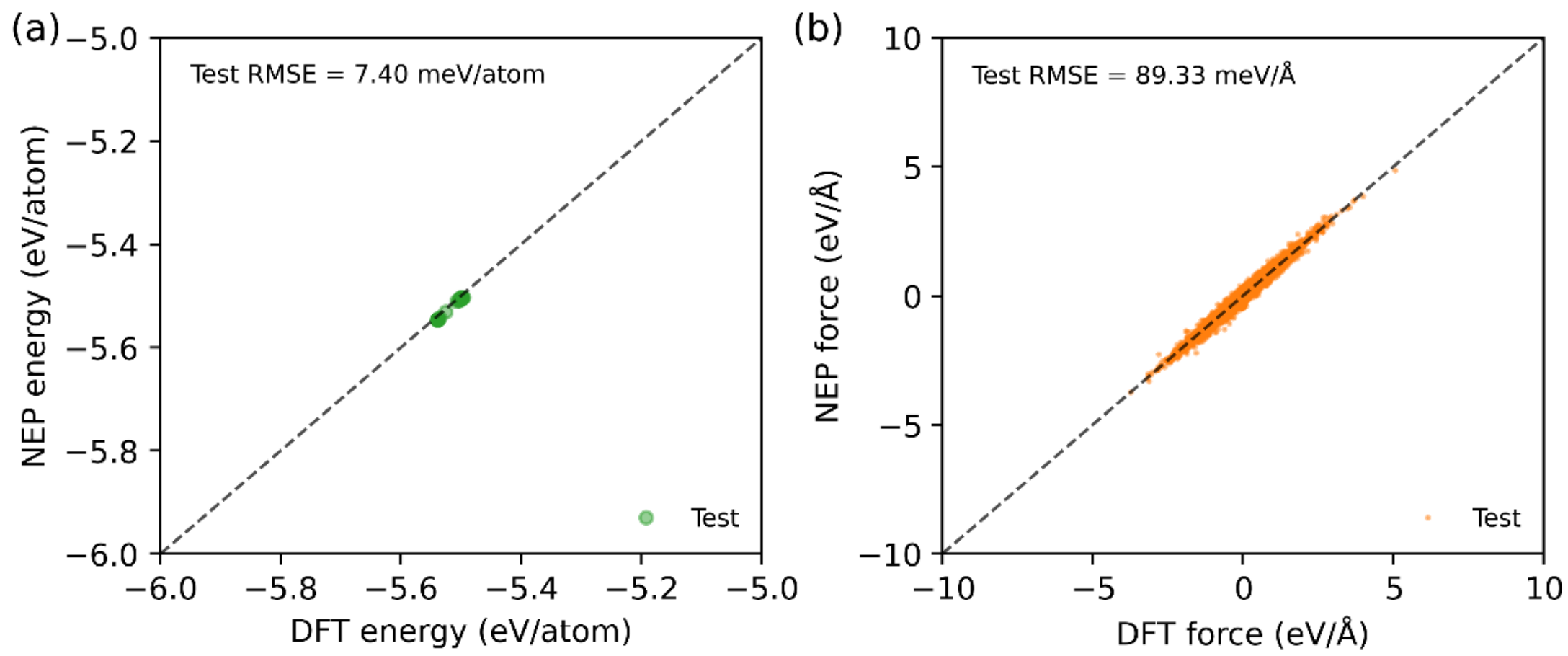

Fig. S2 Validation of the NEP model for [Ch][β-Ala]. Parity plots comparing NEP predictions with reference DFT calculations on the independent test set: (a) energies and (b) atomic forces.

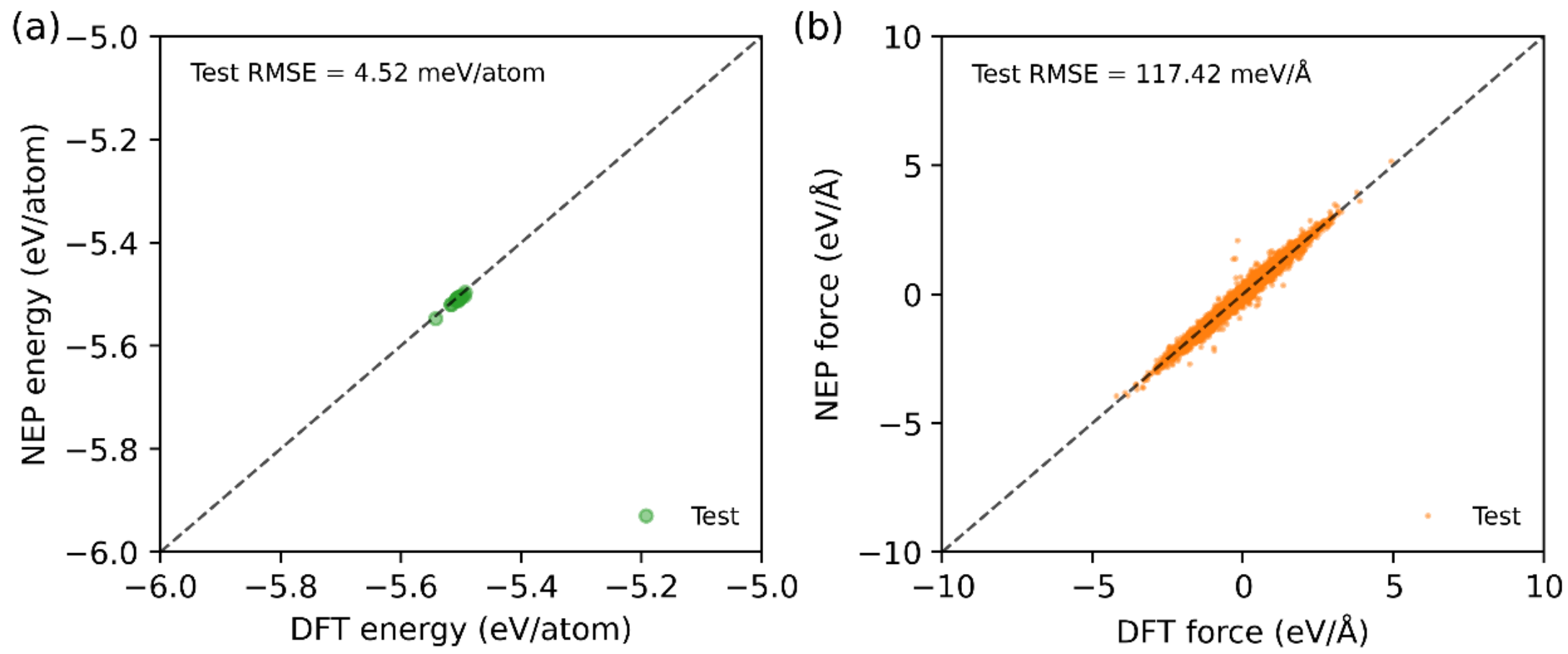

Fig. S3 Validation of the NEP model for [Ch][Gly]. Parity plots comparing NEP predictions with reference DFT calculations on the independent test set: (a) energies and (b) atomic forces.

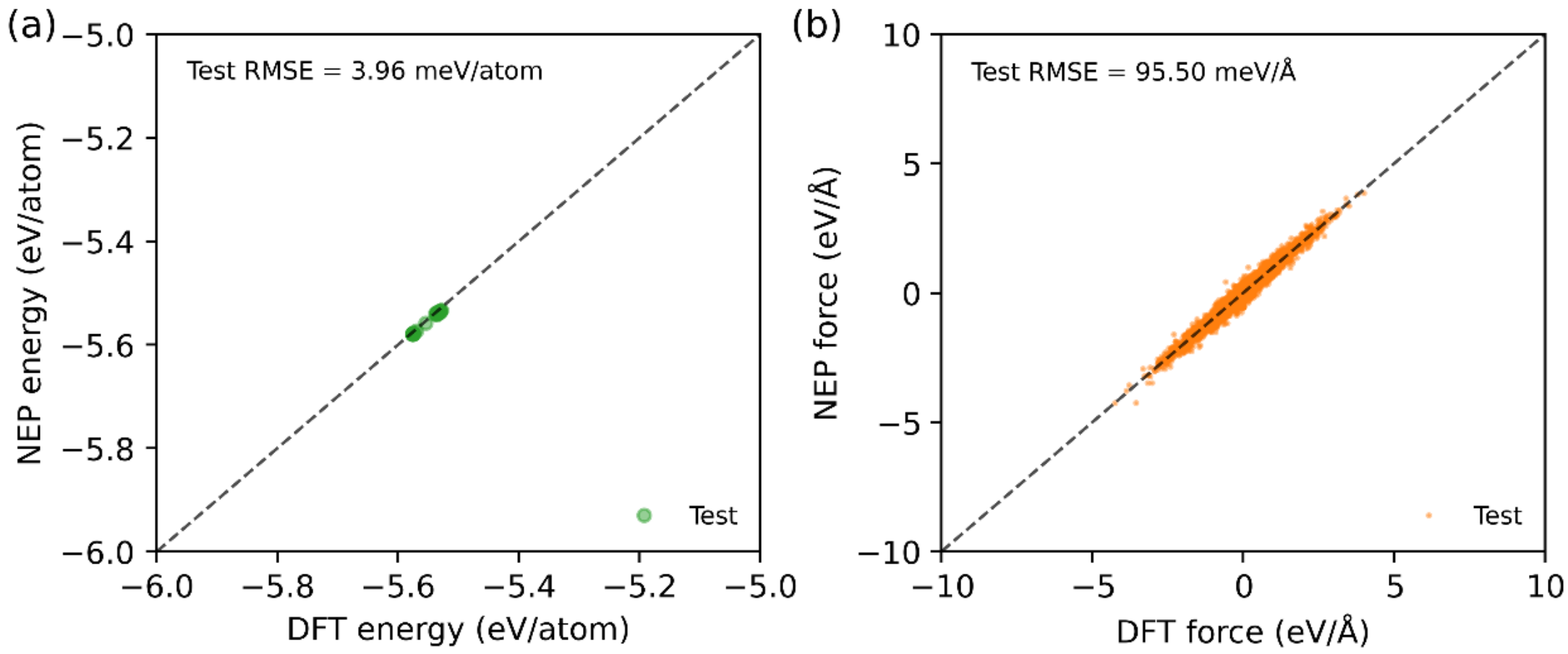

Fig. S4 Validation of the NEP model for [Ch][Ser]. Parity plots comparing NEP predictions with reference DFT calculations on the independent test set: (a) energies and (b) atomic forces.

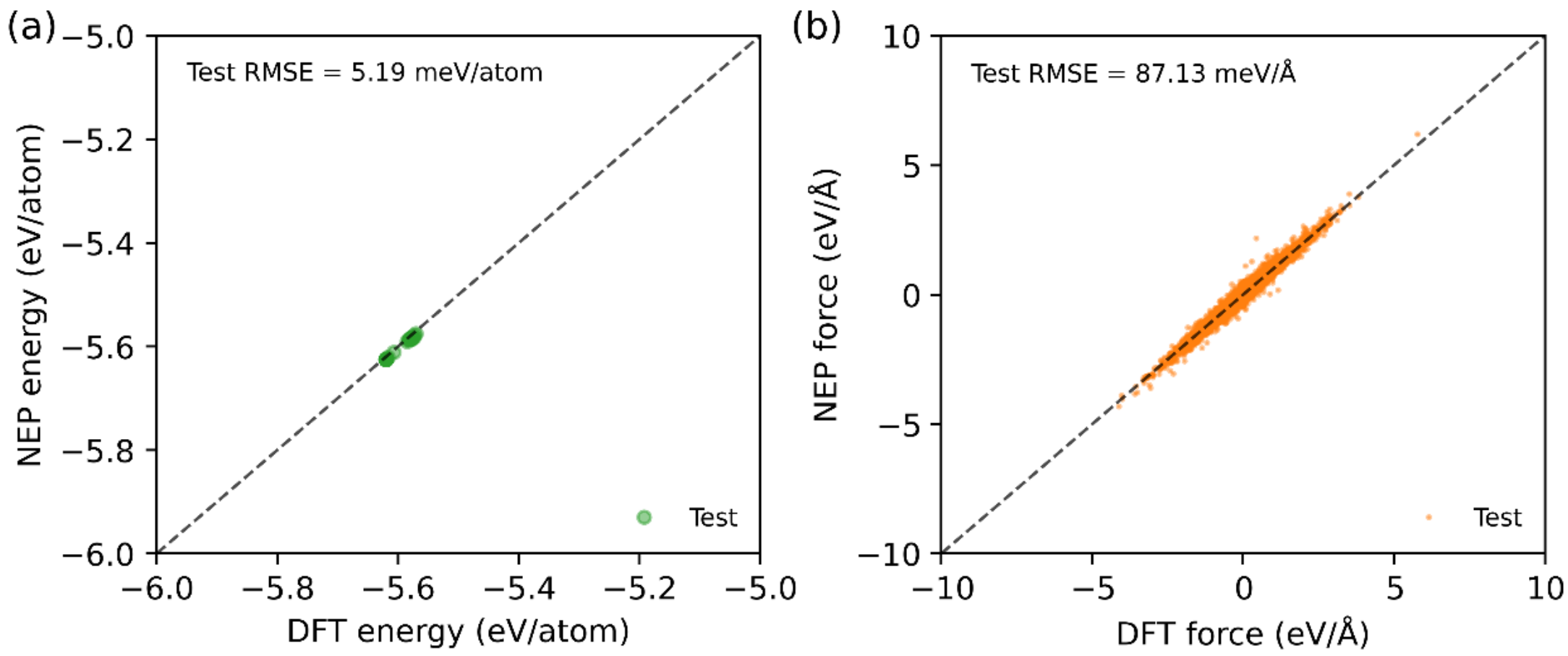

Fig. S5 Validation of the NEP model for [Ch][Pro]. Parity plots comparing NEP predictions with reference DFT calculations on the independent test set: (a) energies and (b) atomic forces.

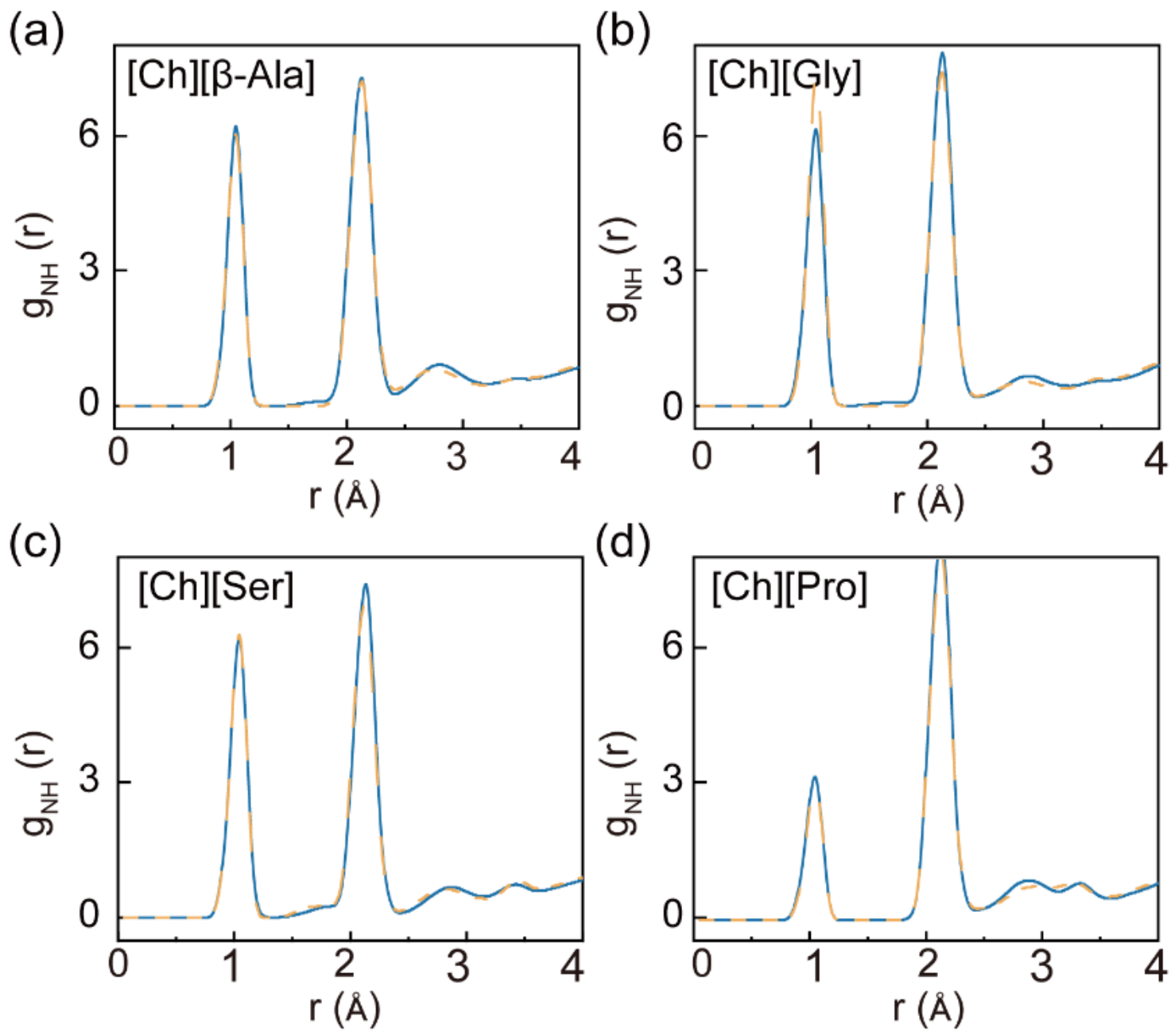

Fig. S6 Comparison of Radial distribution functions (RDFs) for key N–H donor-acceptor pairs. RDFs of key N–H donor-acceptor pairs obtained from NEP-based molecular dynamics simulations (NEP-MD) are compared with those from short DFT simulations for (a) [Ch][*β*-Ala], (b) [Ch][Gly], (c) [Ch][Ser], and (d) [Ch][Pro]. Solid and dashed lines denote NEP-MD and DFT results, respectively.

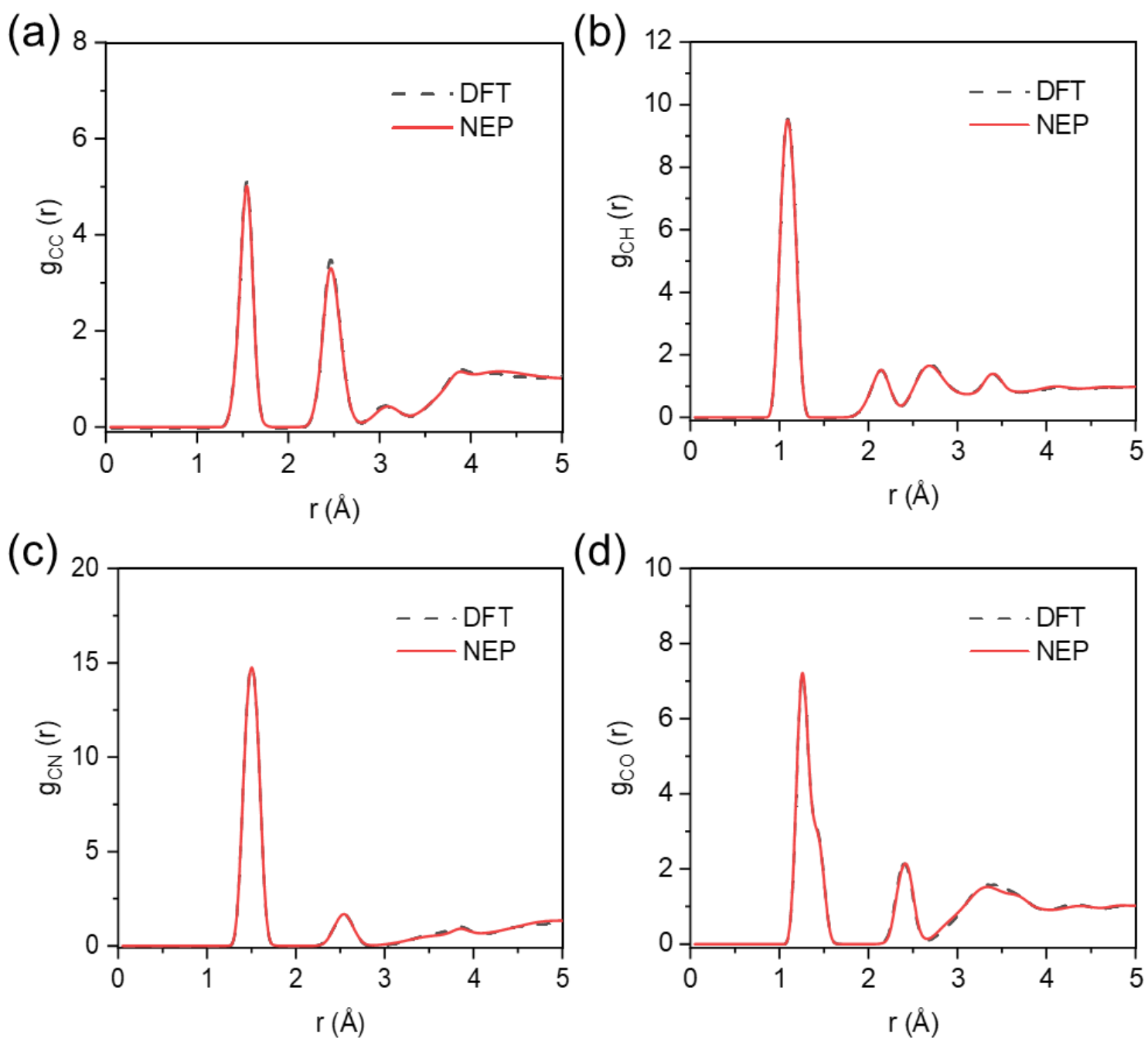

Fig. S7 Comparison of RDFs for selected atom pairs in [Ch][*β*-Ala]. RDFs of selected atom pairs obtained from NEP-MD trajectories and DFT simulations: (a) C–C, (b) C–H, (c) C–N,

and (d) C–O. Solid and dashed lines denote NEP-MD and DFT results, respectively.

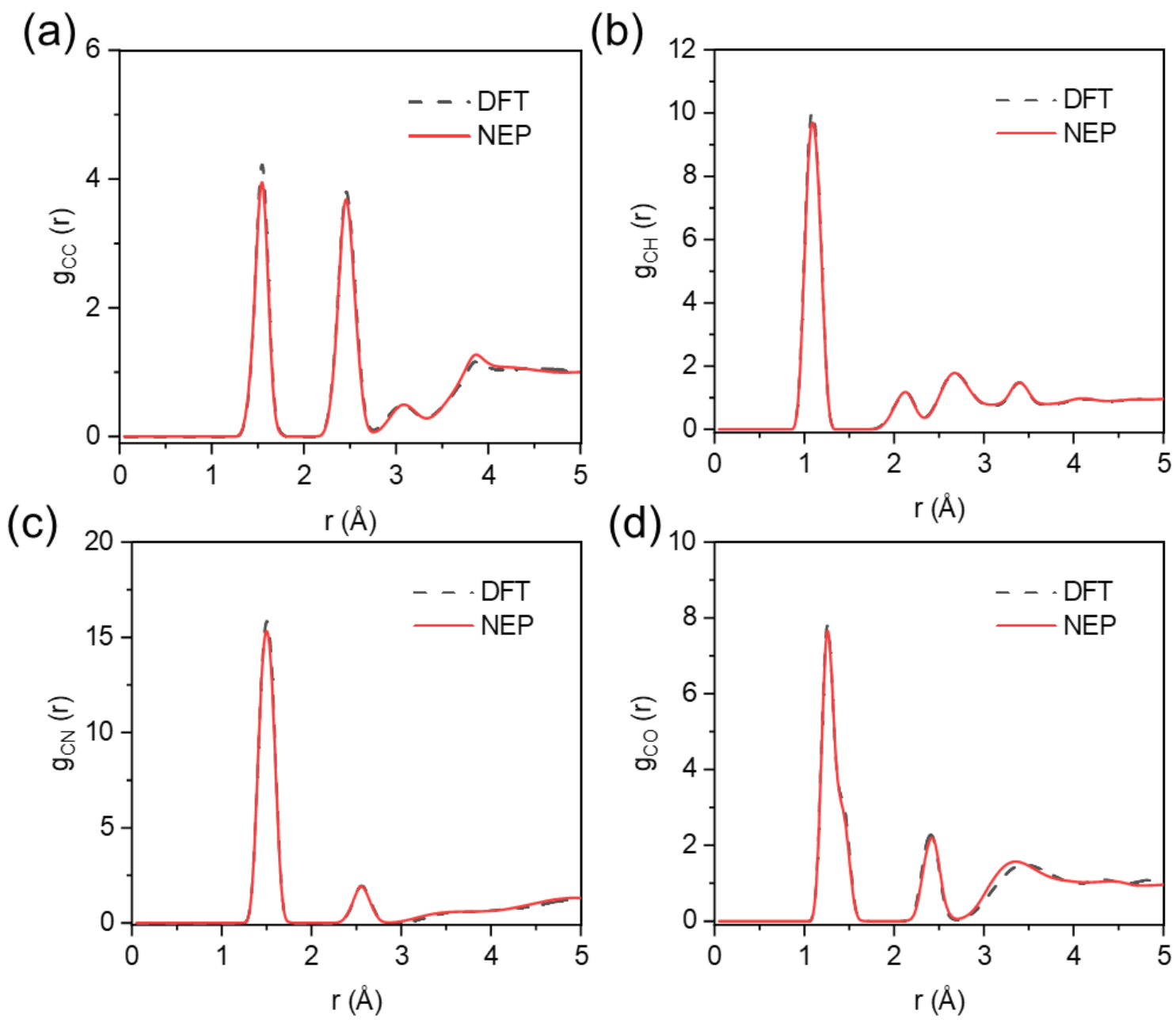

Fig. S8 Comparison of RDFs for selected atom pairs in [Ch][Gly]. RDFs of selected atom pairs obtained from NEP-MD trajectories and DFT simulations: (a) C–C, (b) C–H, (c) C–N, and (d) C–O. Solid and dashed lines denote NEP-MD and DFT results, respectively.

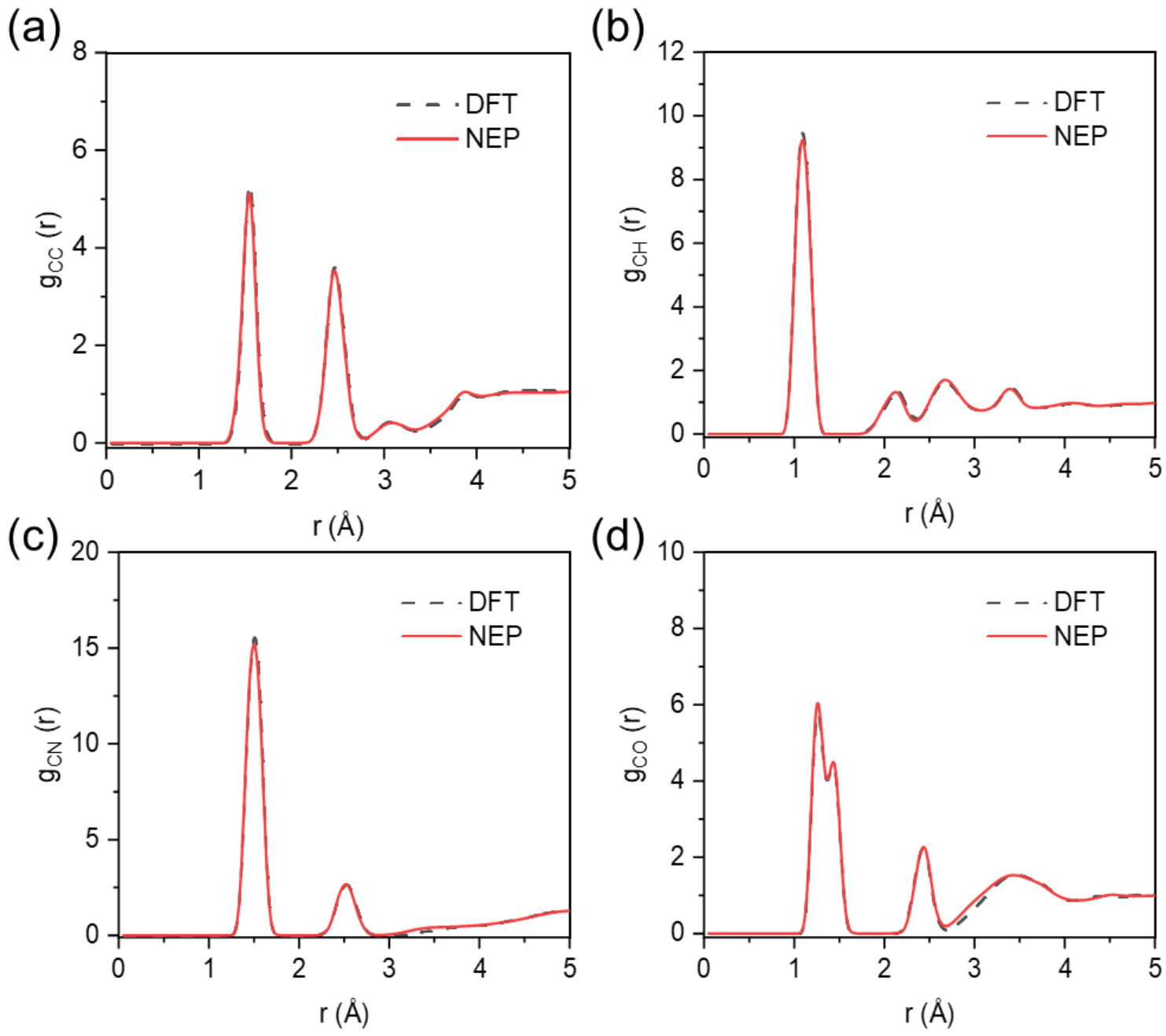

Fig. S9 Comparison of RDFs for selected atom pairs in [Ch][Ser]. RDFs of selected atom pairs obtained from NEP-MD trajectories and DFT simulations: (a) C–C, (b) C–H, (c) C–N, and (d) C–O. Solid and dashed lines denote NEP-MD and DFT results, respectively.

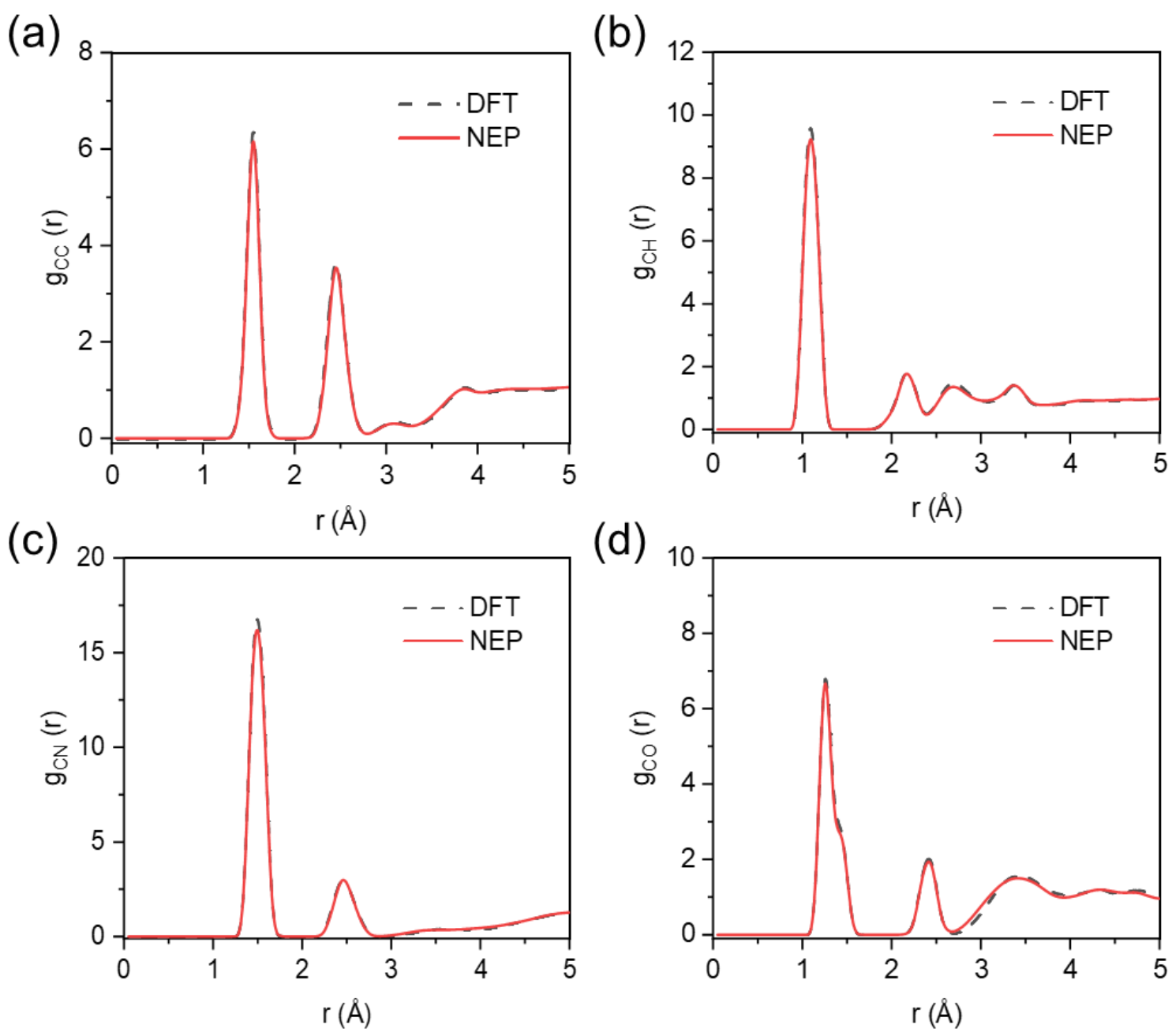

Fig. S10 Comparison of RDFs for selected atom pairs in [Ch][Pro]. RDFs of selected atom pairs obtained from NEP-MD trajectories and DFT simulations: (a) C–C, (b) C–H, (c) C–N, and (d) C–O. Solid and dashed lines denote NEP-MD and DFT results, respectively.

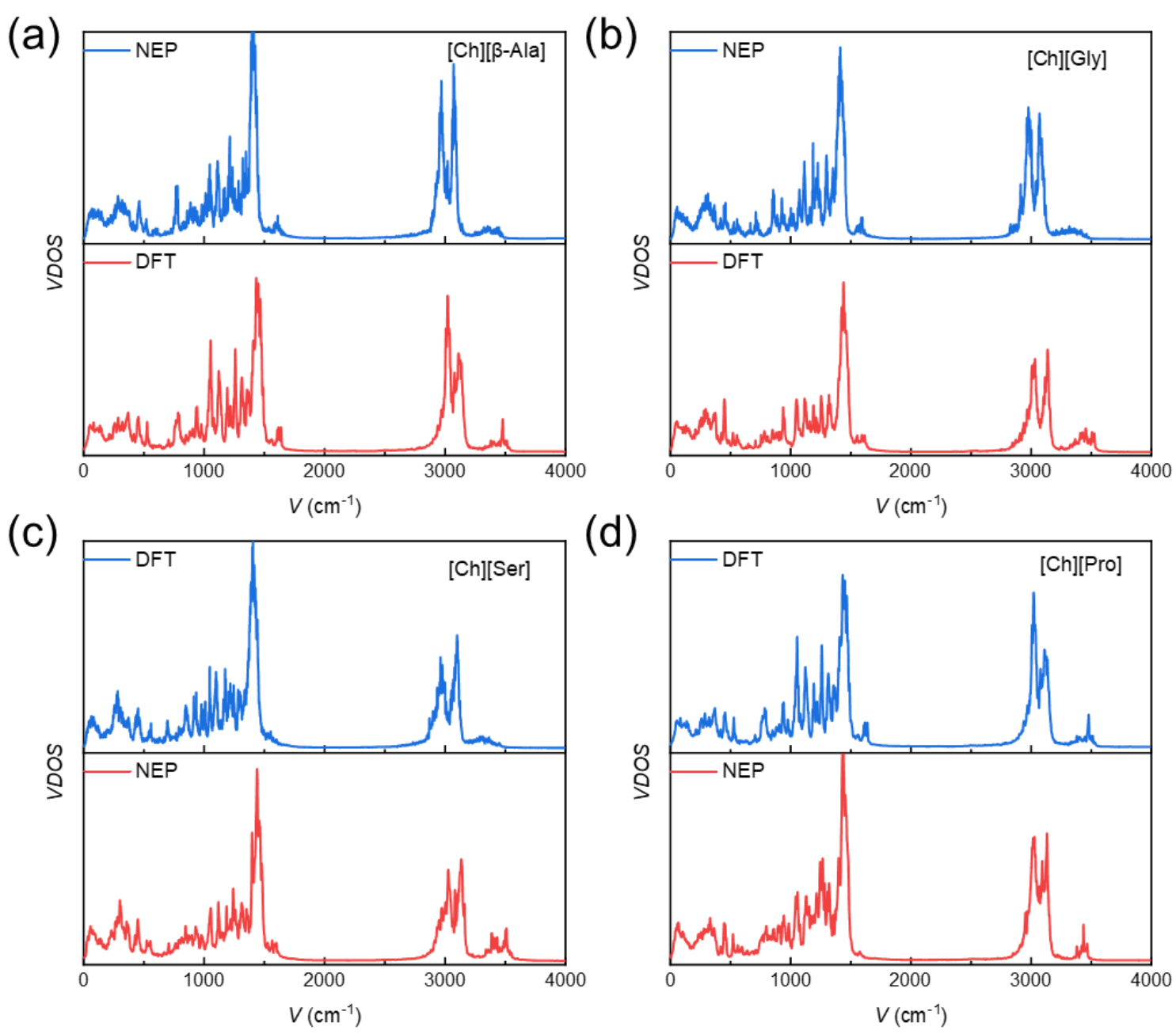

Fig. S11 Vibrational densities of states (VDOS) of the four Ch-AAILs. VDOS calculated from NEP-MD trajectories for (a) [Ch][*β*-Ala], (b) [Ch][Gly], (c) [Ch][Ser], and (d) [Ch][Pro].

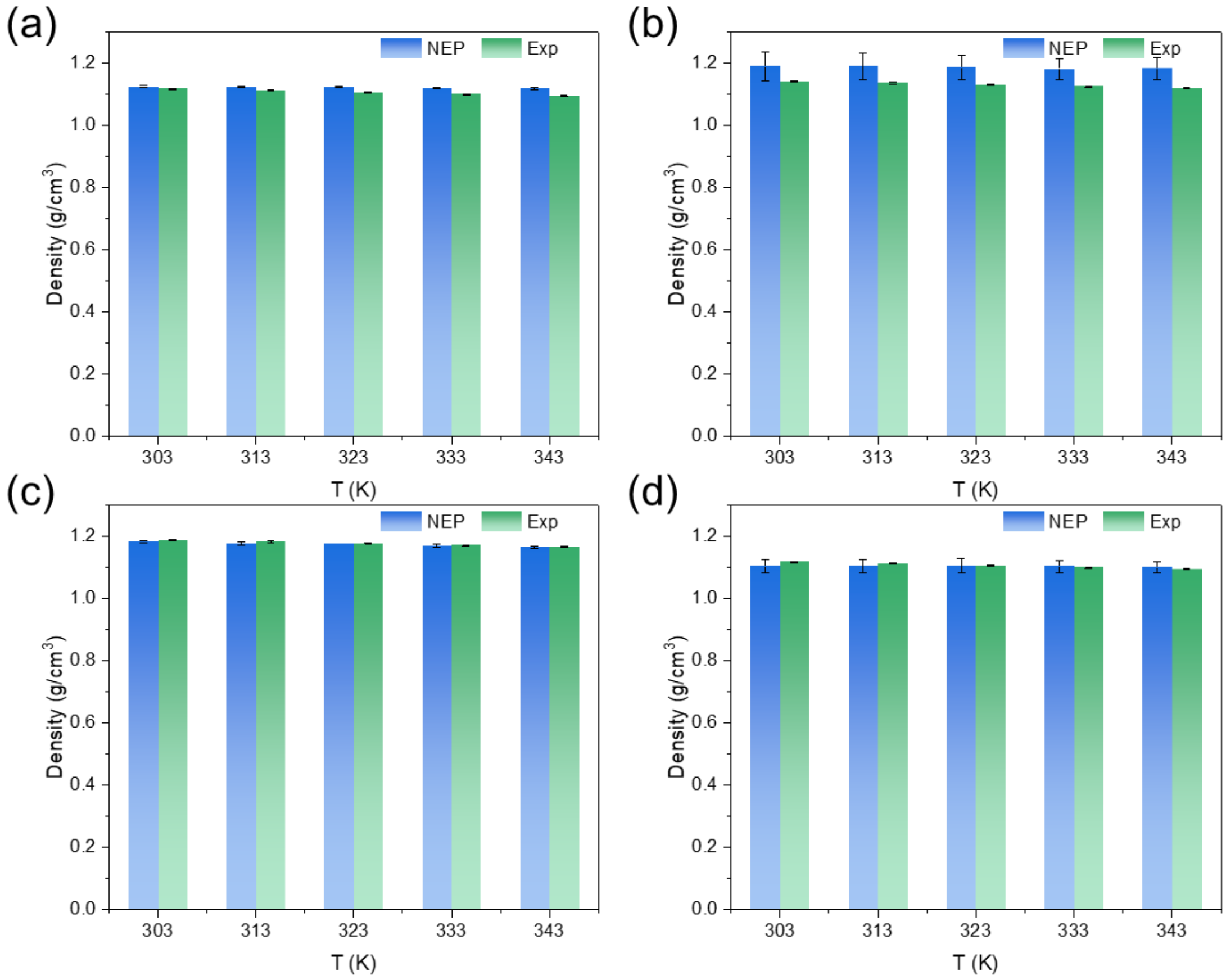

Fig. S12 Temperature-dependent density validation. Comparison of simulated densities from NEP-MD with experimental values as a function of temperature for (a) [Ch][*β*-Ala], (b) [Ch][Gly], (c) [Ch][Ser], and (d) [Ch][Pro].

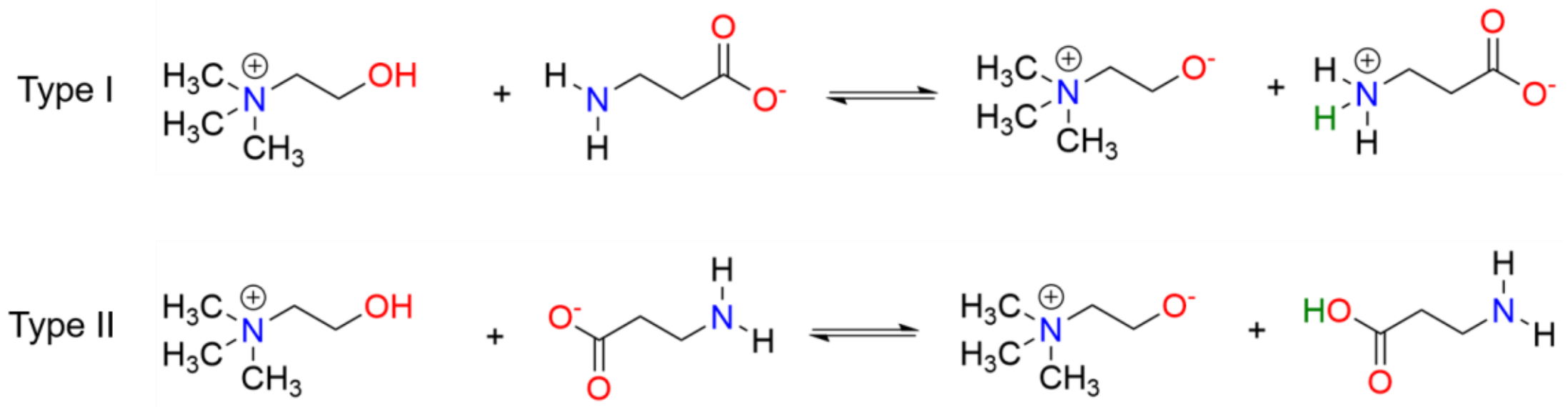

Fig. S13 Primary proton-transfer pathways in Ch-AAILs. Schematic illustration of the two primary local proton-transfer pathways identified in Ch-AAILs: (i) reversible transfer of the cationic hydroxyl proton to the anionic amino group, OH ↔ NH_2, forming zwitterionic cation–anion pairs; and (ii) reversible transfer of the cationic hydroxyl proton to the anionic carbonyl oxygen, OH ↔ O, generating a zwitterionic choline species and a neutral amino acid anion.

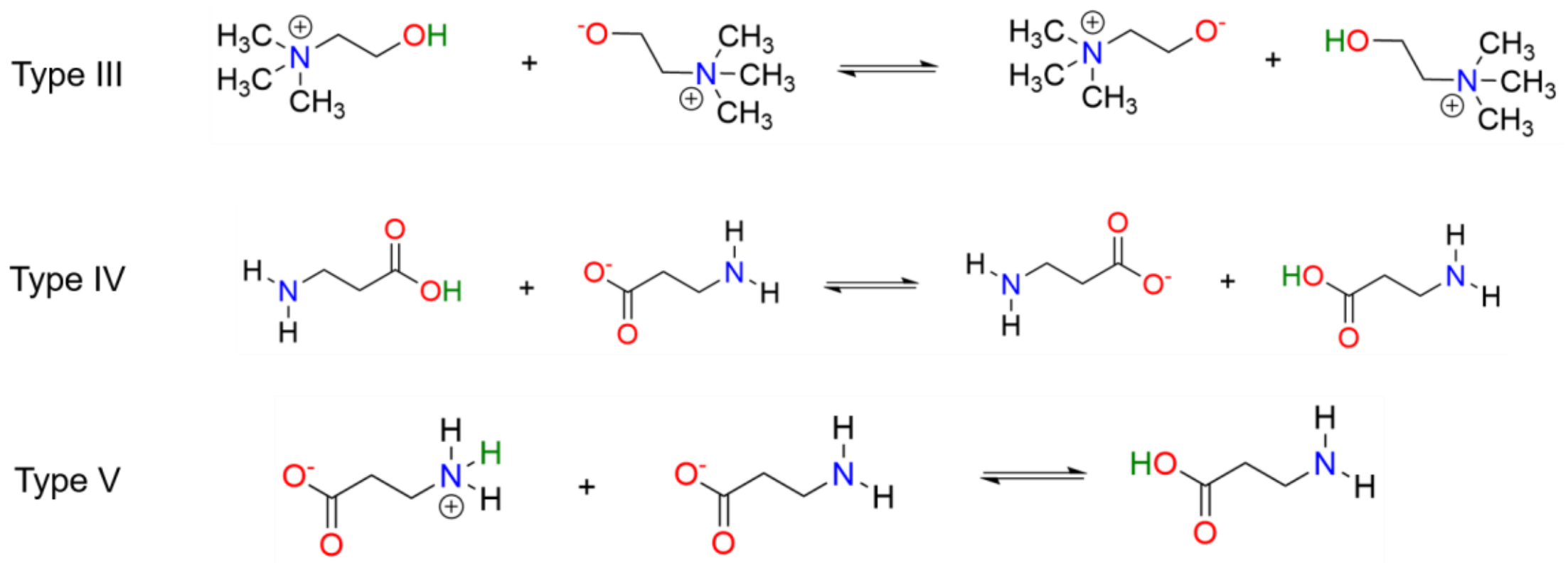

Fig. S14 Secondary local proton-transfer and relay processes. Representative secondary processes following the primary proton-transfer events: (i) localized proton exchange between a normal cation and a zwitterionic cation, O–H···O ↔ O···H–O; (ii) localized proton exchange between a normal anion and a zwitterionic anion, O–H···O ↔ O···H–O; and (iii) intramolecular proton migration within the anion, NH_2–H···O ↔ NH_2···H–O. These processes illustrate local exchange, back-transfer, and short-range proton-relay behavior rather than a fully concerted classical Grotthuss hopping sequence.

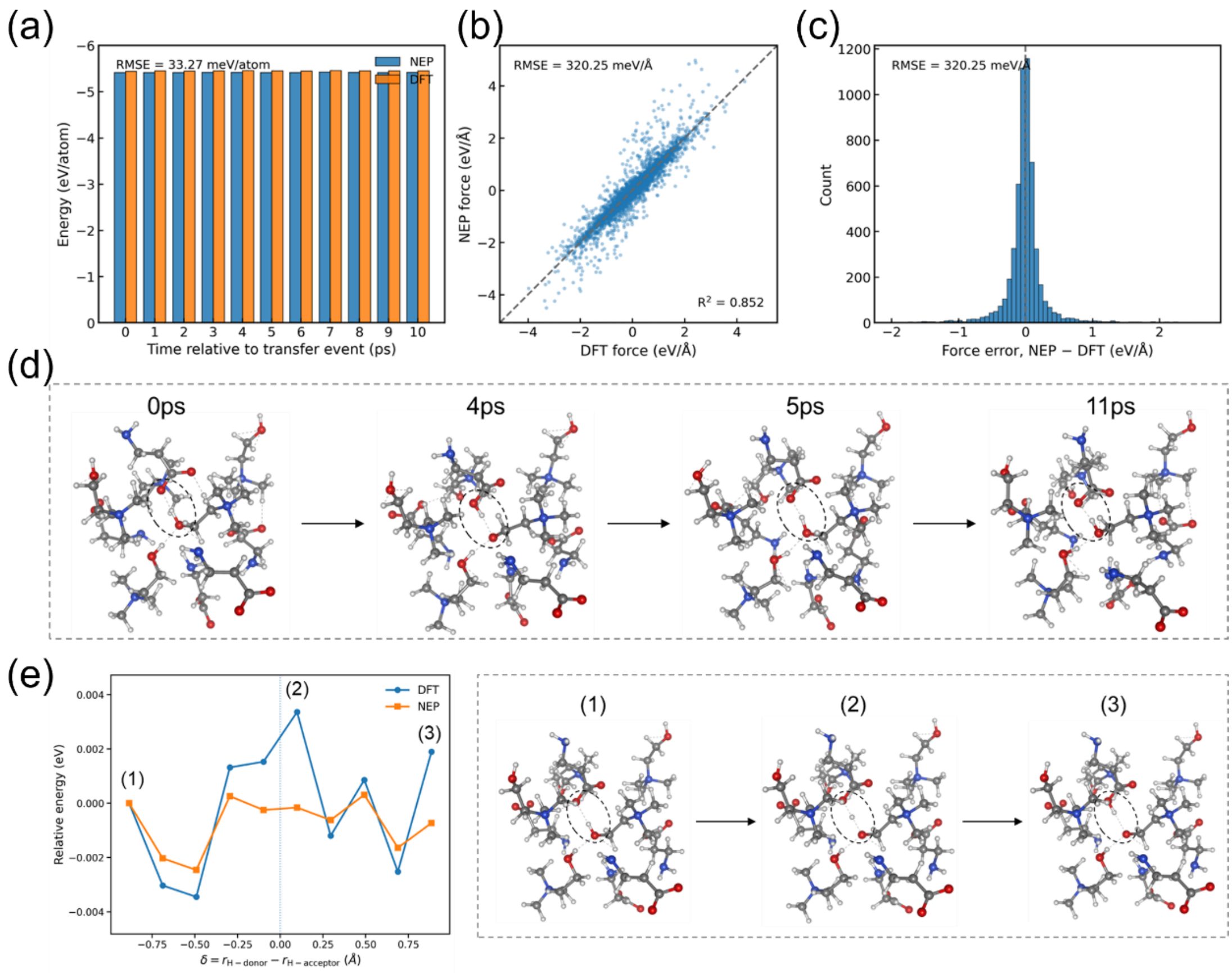

Fig. S15 Validation of NEP predictions for proton-transfer-centered local clusters and representative reaction configurations of [Ch][β-Ala] for the cationic OH ↔ anionic O pathway. Quantitative comparison between NEP and DFT calculations for local cluster configurations extracted from a window spanning 5 ps before to 5 ps after a representative proton-transfer event. (a) Comparison of absolute energies predicted by NEP and DFT. (b) Parity plot of all Cartesian force components from NEP and DFT. (c) Distribution of force errors, defined as $F_{\mathrm{NEP}} - F_{\mathrm{DFT}}$, centered close to zero. (d) Representative cluster structures at selected time points along the transfer event; dashed circles highlight the proton-transfer region. (e) Relative energy profiles along the proton-transfer coordinate, $\delta = r_{\mathrm{H-donor}} - r_{\mathrm{H-acceptor}}$, obtained from NEP and DFT calculations. Key points along the reaction pathway are labeled (1)–(3), with the corresponding representative configurations shown to illustrate the structural evolution during proton migration.

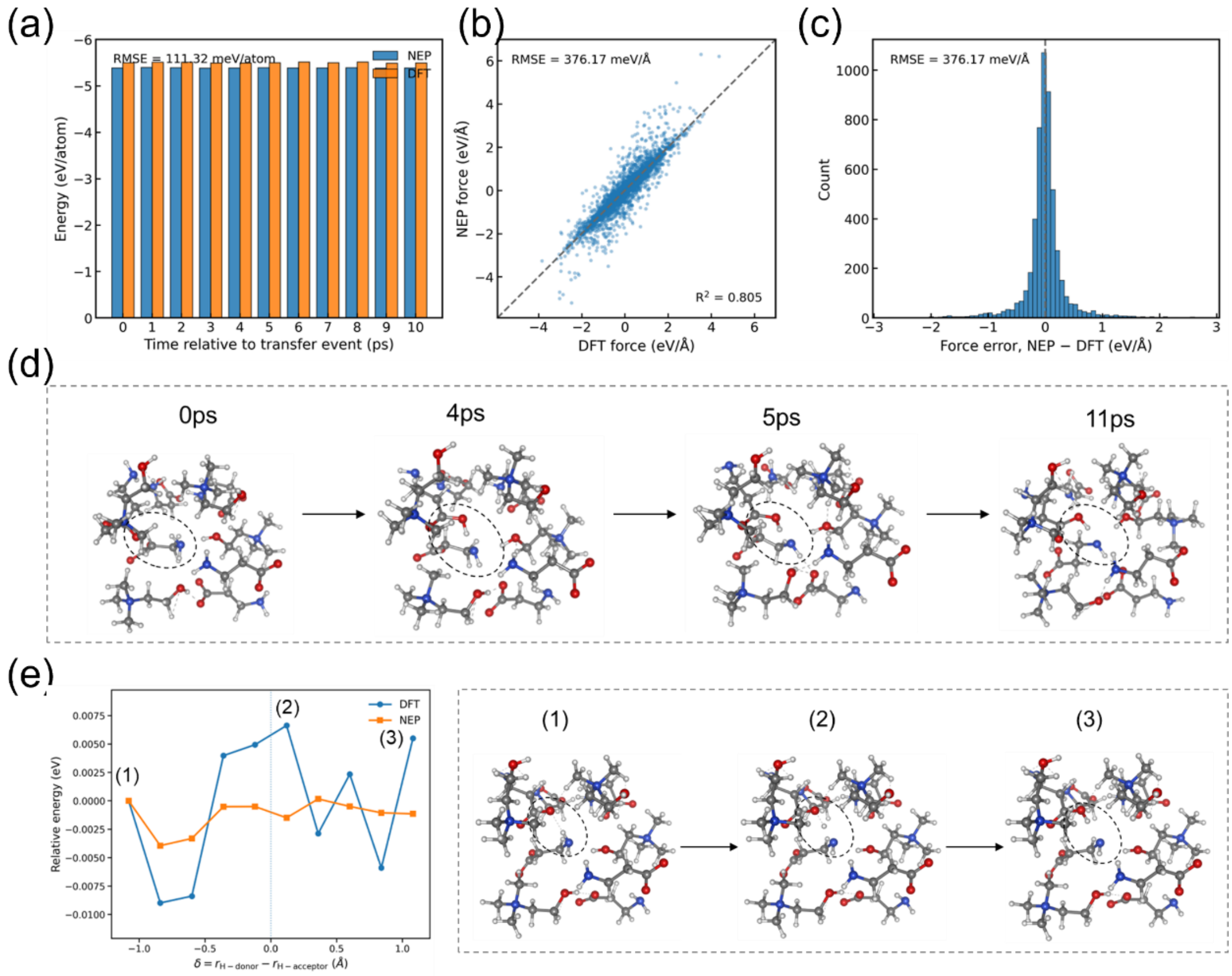

Fig. S16 Validation of NEP predictions for proton-transfer-centered local clusters and representative reaction configurations of [Ch][β-Ala] for the anionic NH_2 ↔ anionic O pathway. Quantitative comparison between NEP and DFT calculations for local cluster configurations extracted from a window spanning 5 ps before to 5 ps after a representative proton-transfer event. (a) Comparison of absolute energies predicted by NEP and DFT. (b) Parity plot of all Cartesian force components from NEP and DFT. (c) Distribution of force errors, defined as $F_{\mathrm{NEP}} - F_{\mathrm{DFT}}$, centered close to zero. (d) Representative cluster structures at selected time points along the transfer event; dashed circles highlight the proton-transfer region. (e) Relative energy profiles along the proton-transfer coordinate, $\delta = r_{\mathrm{H-donor}} - r_{\mathrm{H-acceptor}}$, obtained from NEP and DFT calculations. Key points along the reaction pathway are labeled (1)–(3), with the corresponding representative configurations shown to illustrate the structural evolution during proton migration.

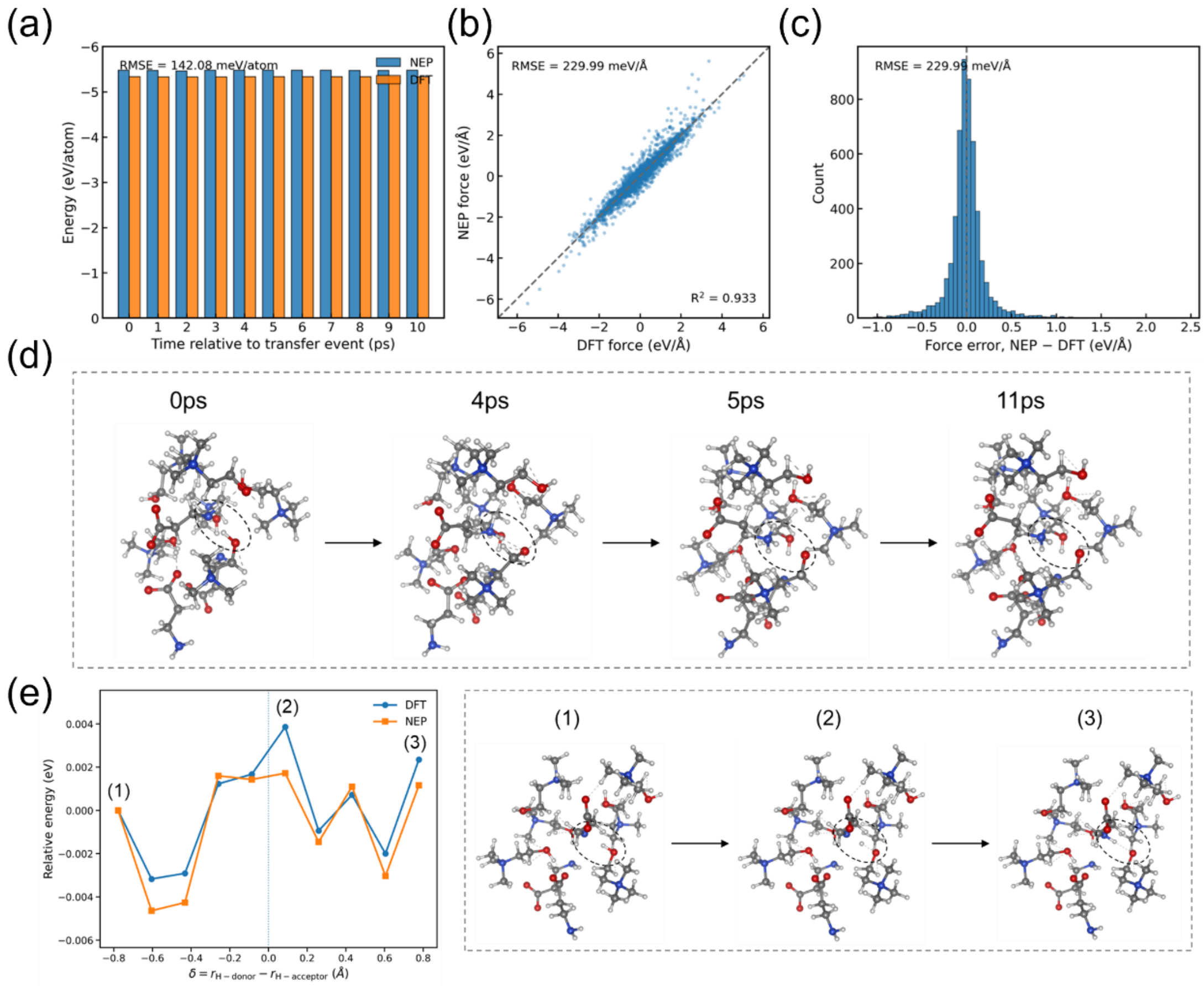

Fig. S17 Validation of NEP predictions for proton-transfer-centered local clusters and representative reaction configurations of [Ch][β-Ala] for the cationic OH ↔ anionic NH_2 pathway. Quantitative comparison between NEP and DFT calculations for local cluster configurations extracted from a window spanning 5 ps before to 5 ps after a representative proton-transfer event. (a) Comparison of absolute energies predicted by NEP and DFT. (b) Parity plot of all Cartesian force components from NEP and DFT. (c) Distribution of force errors, defined as $F_{\mathrm{NEP}} - F_{\mathrm{DFT}}$, centered close to zero. (d) Representative cluster structures at selected time points along the transfer event; dashed circles highlight the proton-transfer region. (e) Relative energy profiles along the proton-transfer coordinate, $\delta = r_{\mathrm{H-donor}} - r_{\mathrm{H-acceptor}}$, obtained from NEP and DFT calculations. Key points along the reaction pathway are labeled (1)–(3), with the corresponding representative configurations shown to illustrate the structural evolution during proton migration.

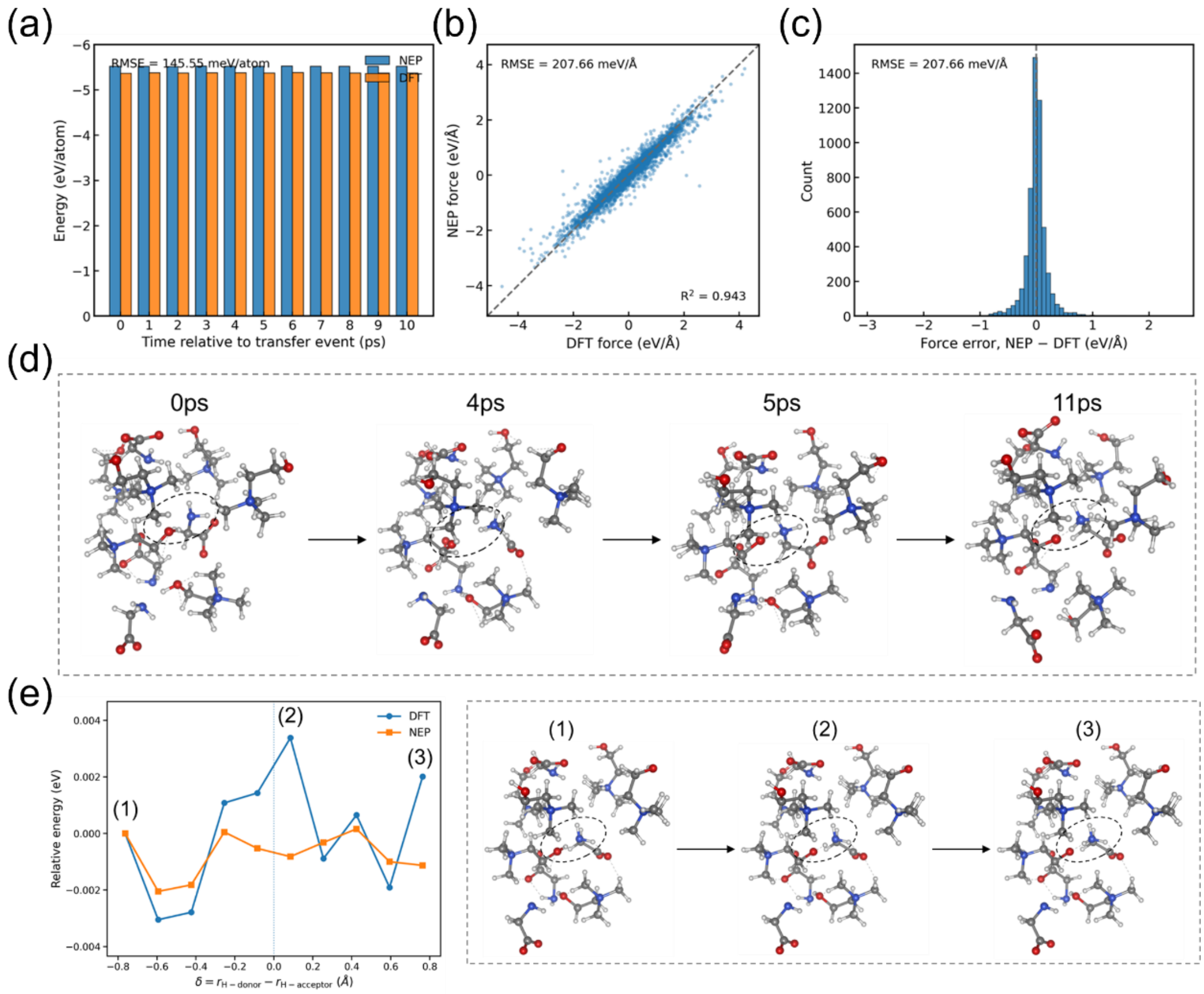

Fig. S18 Validation of NEP predictions for proton-transfer-centered local clusters and representative reaction configurations of [Ch][Gly] for the cationic OH ↔ anionic NH_2 pathway. Quantitative comparison between NEP and DFT calculations for local cluster configurations extracted from extracted from a window spanning 5 ps before to 5 ps after a representative proton-transfer event. (a) Comparison of absolute energies predicted by NEP and DFT. (b) Parity plot of all Cartesian force components from NEP and DFT. (c) Distribution of force errors, defined as $F_{\mathrm{NEP}} - F_{\mathrm{DFT}}$, centered close to zero. (d) Representative cluster structures at selected time points along the transfer event; dashed circles highlight the proton-transfer region. (e) Relative energy profiles along the proton-transfer coordinate, $\delta = r_{\mathrm{H-donor}} - r_{\mathrm{H-acceptor}}$, obtained from NEP and DFT calculations. Key points along the reaction pathway are labeled (1)–(3), with the corresponding representative configurations shown to illustrate the structural evolution during proton migration.

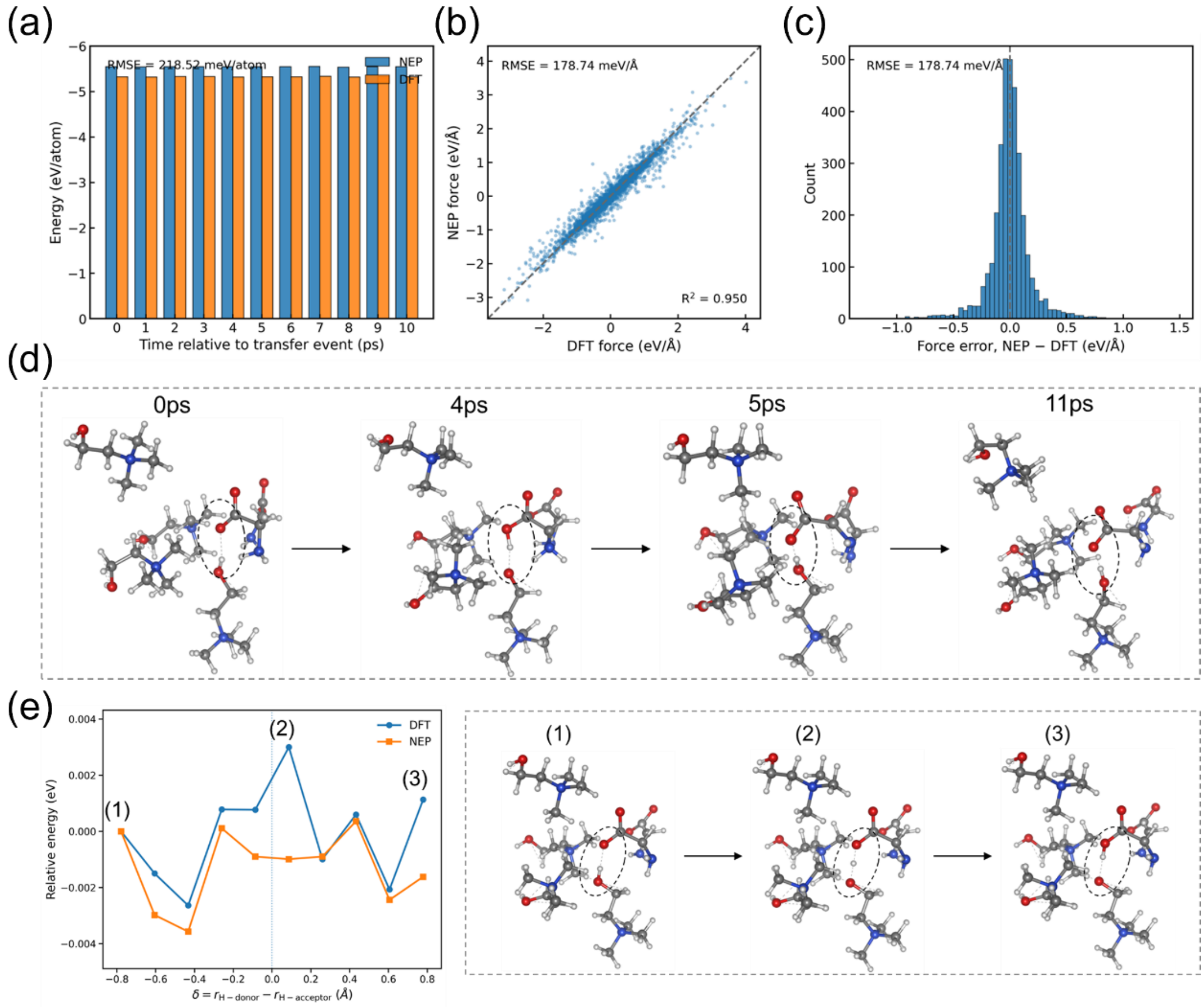

Fig. S19 Validation of NEP predictions for proton-transfer-centered local clusters and representative reaction configurations of [Ch][Gly] for the cationic OH ↔ anionic O pathway. Quantitative comparison between NEP and DFT calculations for local cluster configurations extracted from a window spanning 5 ps before to 5 ps after a representative proton-transfer event. (a) Comparison of absolute energies predicted by NEP and DFT. (b) Parity plot of all Cartesian force components from NEP and DFT. (c) Distribution of force errors, defined as $F_{\mathrm{NEP}} - F_{\mathrm{DFT}}$, centered close to zero. (d) Representative cluster structures at selected time points along the transfer event; dashed circles highlight the proton-transfer region. (e) Relative energy profiles along the proton-transfer coordinate, $\delta = r_{\mathrm{H-donor}} - r_{\mathrm{H-acceptor}}$, obtained from NEP and DFT calculations. Key points along the reaction pathway are labeled (1)–(3), with the corresponding representative configurations shown to illustrate the structural evolution during proton migration.

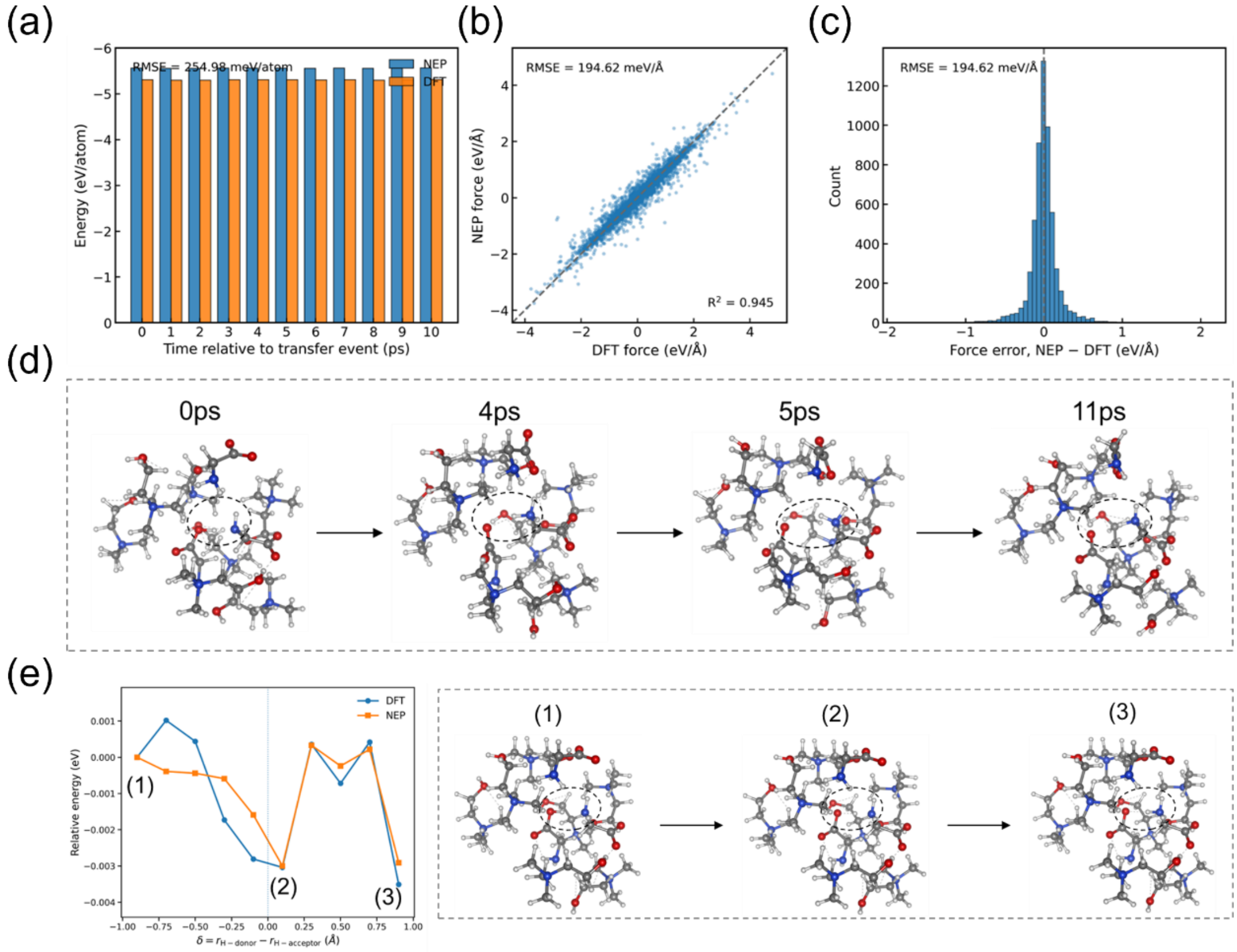

Fig. S20 Validation of NEP predictions for proton-transfer-centered local clusters and representative reaction configurations of [Ch][Gly] for the anionic OH ↔ anionic NH_2 pathway. Quantitative comparison between NEP and DFT calculations for local cluster configurations extracted from a window spanning 5 ps before to 5 ps after a representative proton-transfer event. (a) Comparison of absolute energies predicted by NEP and DFT. (b) Parity plot of all Cartesian force components from NEP and DFT. (c) Distribution of force errors, defined as $F_{\mathrm{NEP}} - F_{\mathrm{DFT}}$, centered close to zero. (d) Representative cluster structures at selected time points along the transfer event; dashed circles highlight the proton-transfer region. (e) Relative energy profiles along the proton-transfer coordinate, $\delta = r_{\mathrm{H-donor}} - r_{\mathrm{H-acceptor}}$, obtained from NEP and DFT calculations. Key points along the reaction pathway are labeled (1)–(3), with the corresponding representative configurations shown to illustrate the structural evolution during proton migration.

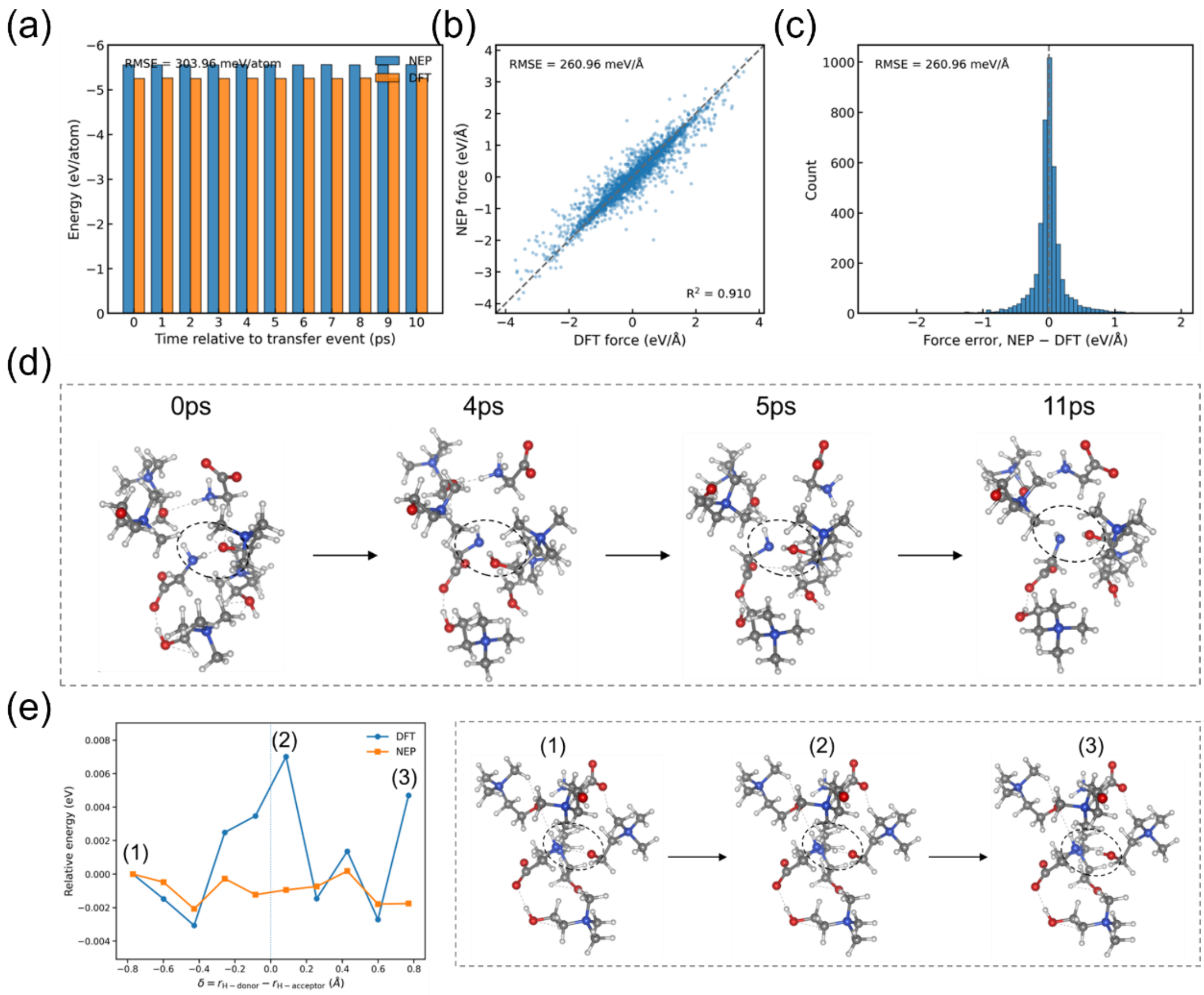

Fig. S21 Validation of NEP predictions for proton-transfer-centered local clusters and representative reaction configurations of [Ch][Gly] for the anionic NH_2 ↔ cationic O pathway. Quantitative comparison between NEP and DFT calculations for local cluster configurations extracted from a window spanning 5 ps before to 5 ps after a representative proton-transfer event. (a) Comparison of absolute energies predicted by NEP and DFT. (b) Parity plot of all Cartesian force components from NEP and DFT. (c) Distribution of force errors, defined as $F_{\mathrm{NEP}} - F_{\mathrm{DFT}}$, centered close to zero. (d) Representative cluster structures at selected time points along the transfer event; dashed circles highlight the proton-transfer region. (e) Relative energy profiles along the proton-transfer coordinate, $\delta = r_{\mathrm{H-donor}} - r_{\mathrm{H-acceptor}}$, obtained from NEP and DFT calculations. Key points along the reaction pathway are labeled (1)–(3), with the corresponding representative configurations shown to illustrate the structural evolution during proton migration.

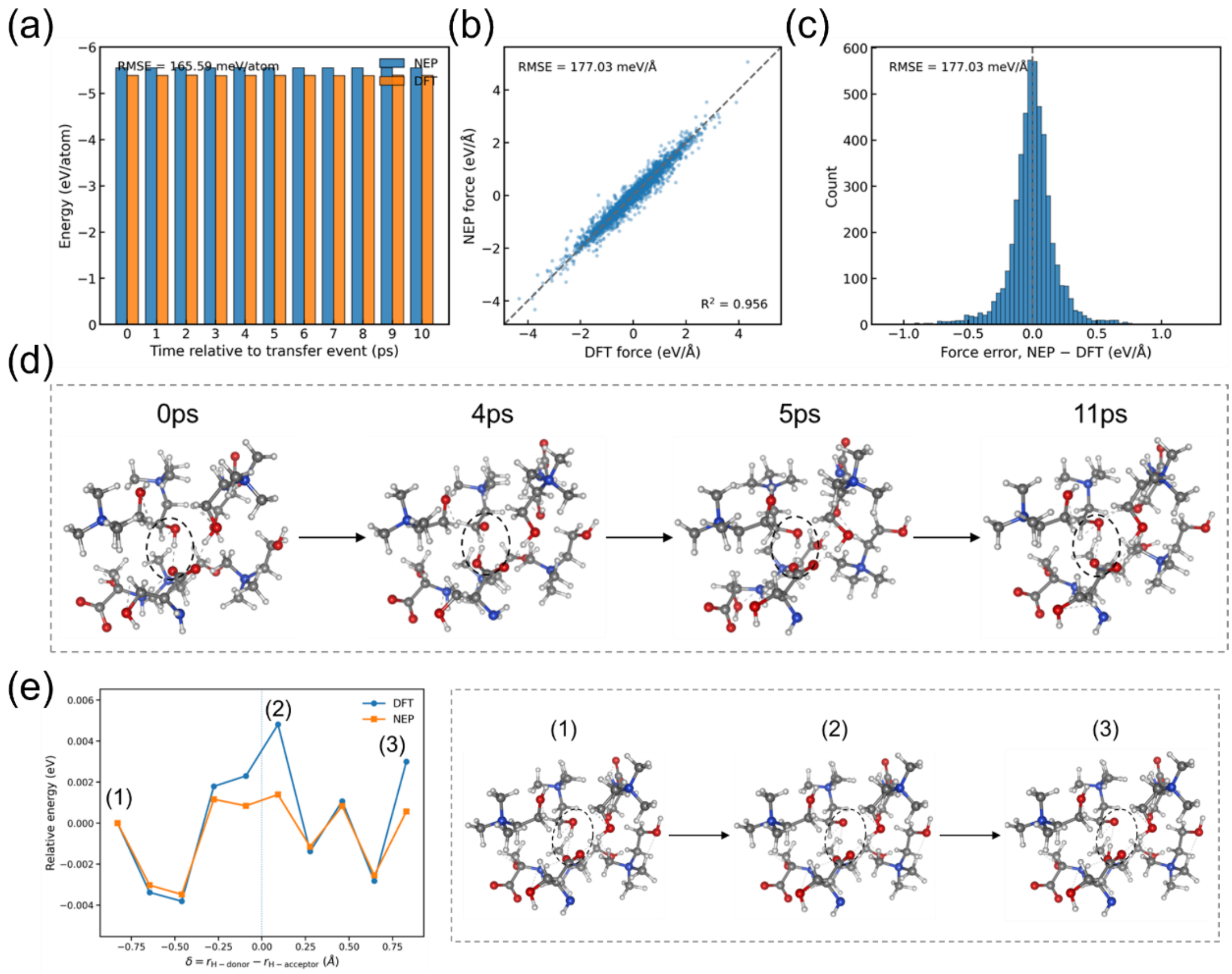

Fig. S22 Validation of NEP predictions for proton-transfer-centered local clusters and representative reaction configurations of [Ch][Ser] for the cationic OH ↔ anionic O pathway. Quantitative comparison between NEP and DFT calculations for local cluster configurations extracted from a window spanning 5 ps before to 5 ps after a representative proton-transfer event. (a) Comparison of absolute energies predicted by NEP and DFT. (b) Parity plot of all Cartesian force components from NEP and DFT. (c) Distribution of force errors, defined as $F_{\mathrm{NEP}} - F_{\mathrm{DFT}}$, centered close to zero. (d) Representative cluster structures at selected time points along the transfer event; dashed circles highlight the proton-transfer region. (e) Relative energy profiles along the proton-transfer coordinate, $\delta = r_{\mathrm{H-donor}} - r_{\mathrm{H-acceptor}}$, obtained from NEP and DFT calculations. Key points along the reaction pathway are labeled (1)–(3), with the corresponding representative configurations shown to illustrate the structural evolution during proton migration.

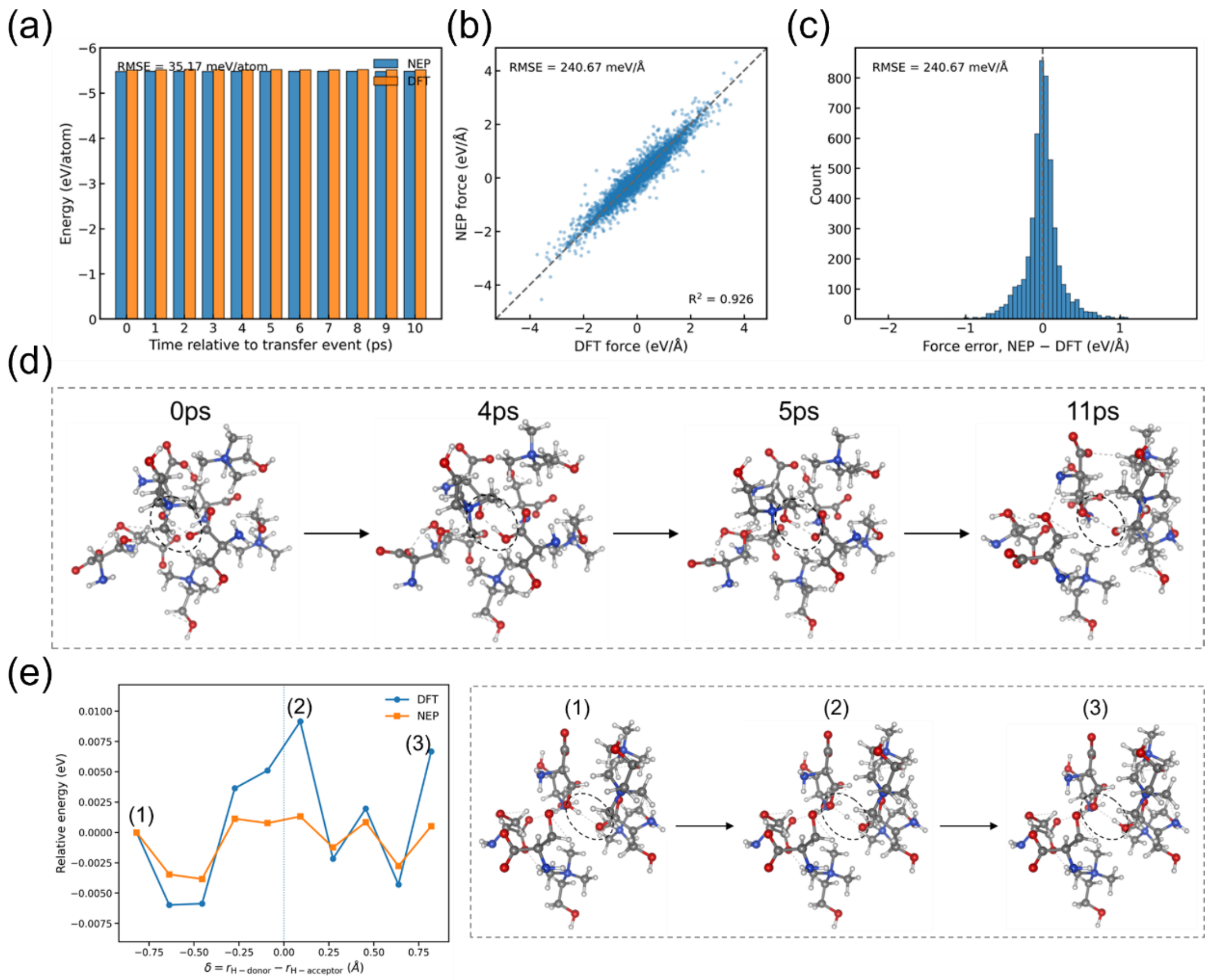

Fig. S23 Validation of NEP predictions for proton-transfer-centered local clusters and representative reaction configurations of [Ch][Ser] for the anionic OH ↔ anionic O pathway. Quantitative comparison between NEP and DFT calculations for local cluster configurations extracted from a window spanning 5 ps before to 5 ps after a representative proton-transfer event. (a) Comparison of absolute energies predicted by NEP and DFT. (b) Parity plot of all Cartesian force components from NEP and DFT. (c) Distribution of force errors, defined as $F_{\mathrm{NEP}} - F_{\mathrm{DFT}}$, centered close to zero. (d) Representative cluster structures at selected time points along the transfer event; dashed circles highlight the proton-transfer region. (e) Relative energy profiles along the proton-transfer coordinate, $\delta = r_{\mathrm{H-donor}} - r_{\mathrm{H-acceptor}}$, obtained from NEP and DFT calculations. Key points along the reaction pathway are labeled (1)–(3), with the corresponding representative configurations shown to illustrate the structural evolution during proton migration.

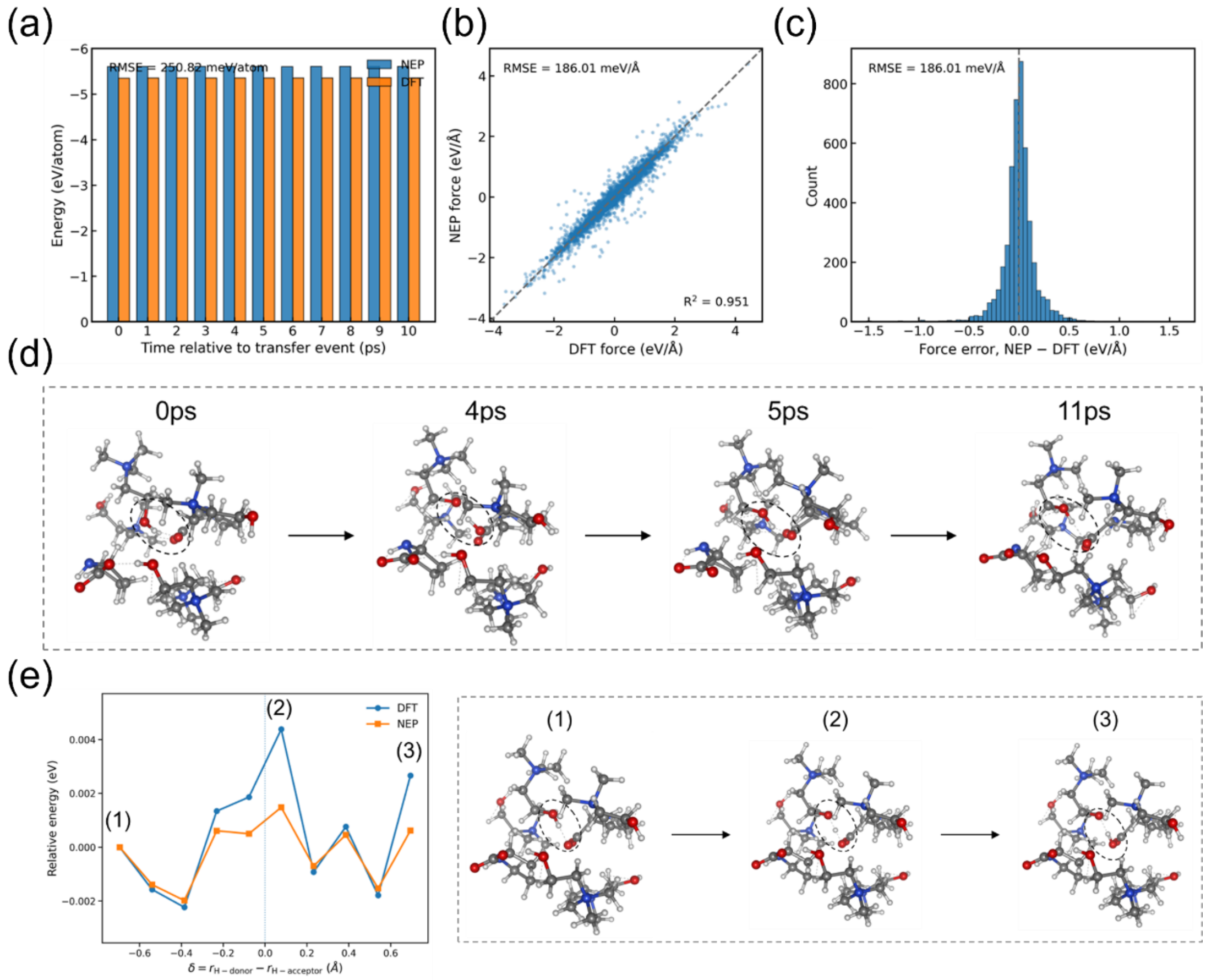

Fig. S24 Validation of NEP predictions for proton-transfer-centered local clusters and representative reaction configurations of [Ch][Pro] for the cationic OH ↔ anionic O pathway. Quantitative comparison between NEP and DFT calculations for local cluster configurations extracted from a window spanning 5 ps before to 5 ps after a representative proton-transfer event. (a) Comparison of absolute energies predicted by NEP and DFT. (b) Parity plot of all Cartesian force components from NEP and DFT. (c) Distribution of force errors, defined as $F_{\mathrm{NEP}} - F_{\mathrm{DFT}}$, centered close to zero. (d) Representative cluster structures at selected time points along the transfer event; dashed circles highlight the proton-transfer region. (e) Relative energy profiles along the proton-transfer coordinate, $\delta = r_{\mathrm{H-donor}} - r_{\mathrm{H-acceptor}}$, obtained from NEP and DFT calculations. Key points along the reaction pathway are labeled (1)–(3), with the corresponding representative configurations shown to illustrate the structural evolution during proton migration.

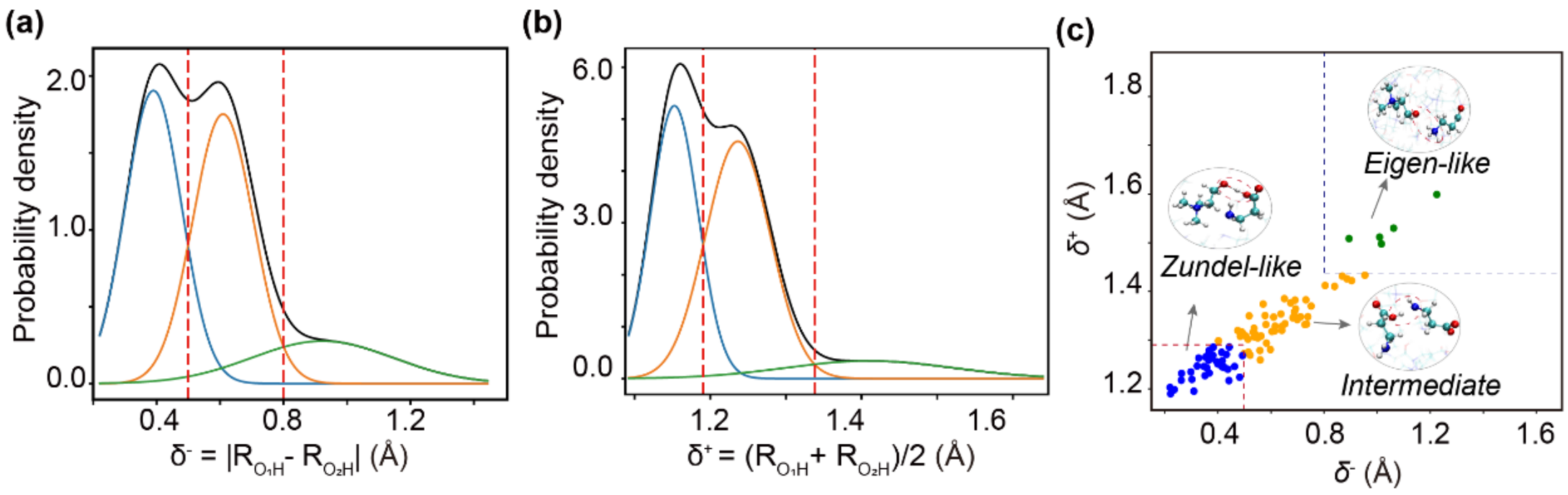

Fig. S25 GMM-assisted classification of transient proton-solvation motifs. (a) One-dimensional Gaussian mixture model (GMM) fit to the asymmetry coordinate, $\delta^- = |R_{O_1H} - R_{O_2H}|$. The total probability density function (black solid line) is decomposed into three components (blue, orange, and green), and the intersections of adjacent components (red dashed lines) define adaptive thresholds separating the Zundel-like, intermediate, and Eigen-like regions. (b) Analogous GMM fit to the average bond-length coordinate, $\delta^+ = (R_{O_1H} + R_{O_2H})/2$, using the same color scheme. (c) Two-dimensional classification based on the thresholds determined in (a) and (b). Configurations with low δ^- and low δ^+ are assigned as Zundel-like, those with high δ^- and high δ^+ as Eigen-like, and the remaining configurations as intermediate.

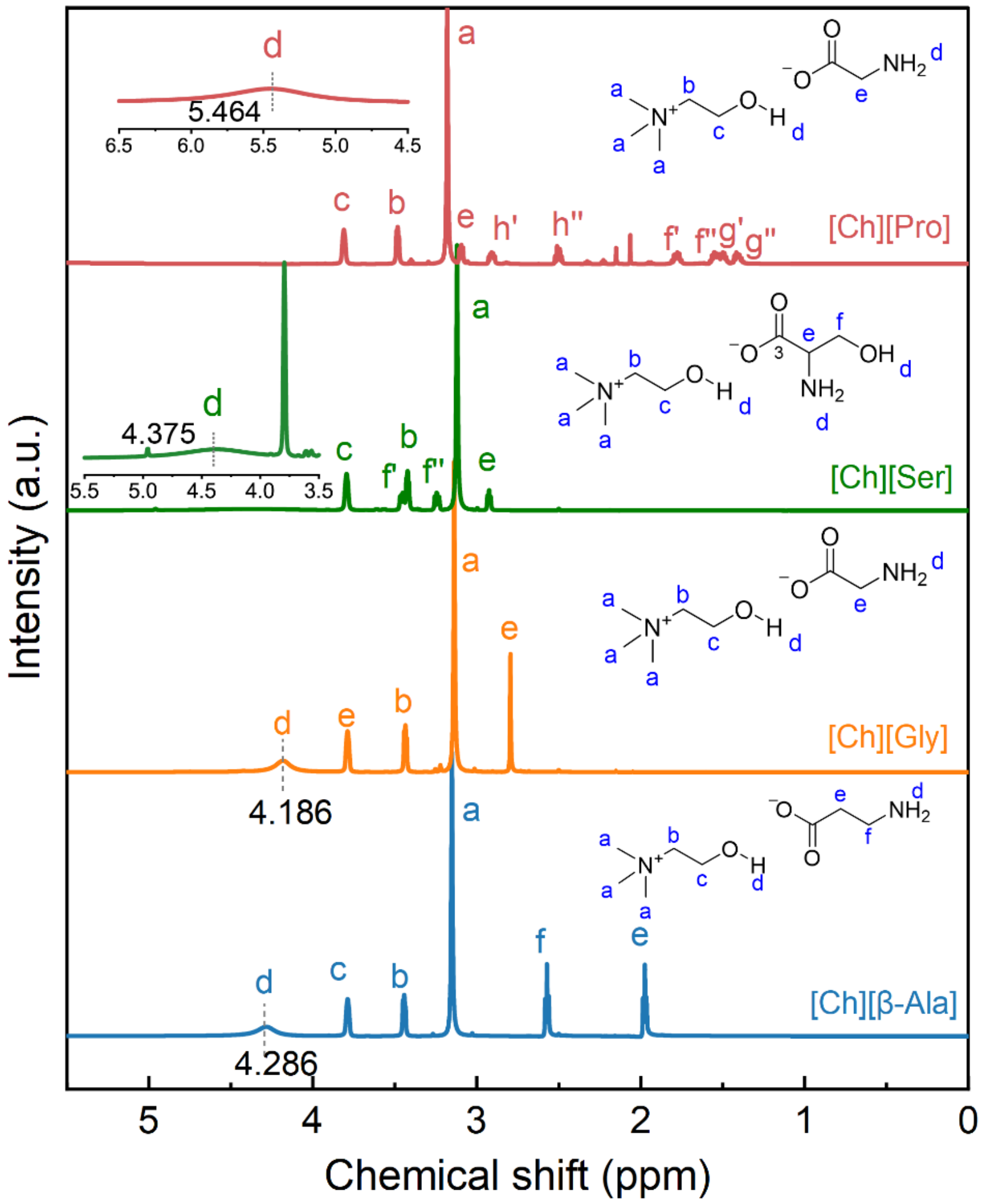

Fig. S26 Normalized and vertically offset ¹H NMR spectra of [Ch][Pro] (top), [Ch][Ser] (second), [Ch][Gly] (third), and [Ch][*β*-Ala] (bottom). Proton resonances are assigned according to the labeled molecular structures (signals a–g). The broad resonance labeled d arises from exchangeable protons, mainly associated with the choline –OH group and amino acid –NH_2 groups, which coalesce into a single averaged signal due to fast exchange on the NMR timescale. The chemical shift of peak d is indicated for each ionic liquid.

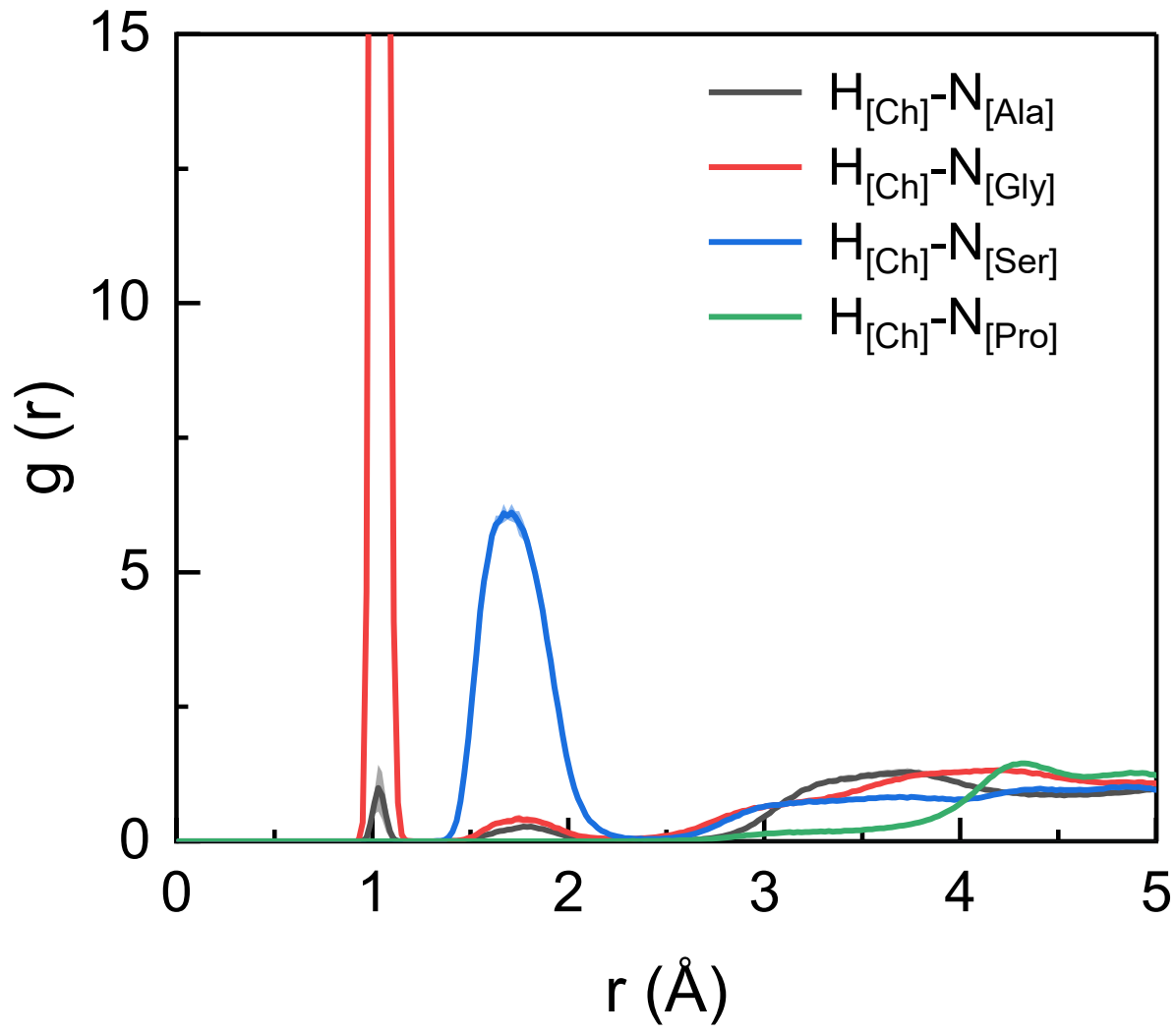

Fig. S27 RDFs between cationic protons and anionic nitrogen atoms. RDFs at 303 K from NEP-MD simulations showing distances between cationic protons and anionic nitrogen atoms for [Ch][β-Ala] (black), [Ch][Gly] (red), [Ch][Ser] (blue), and [Ch][Pro] (green).

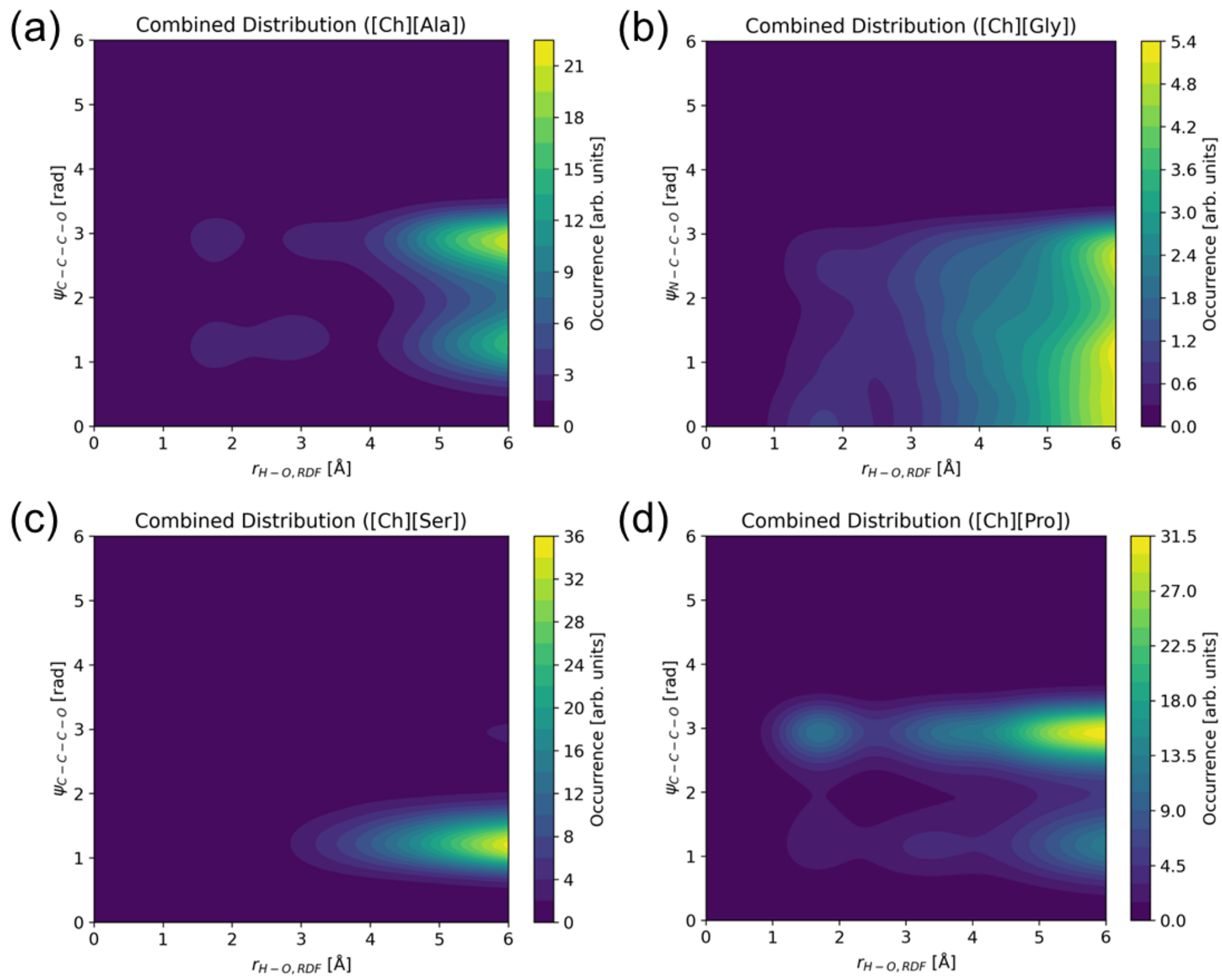

Fig. S28 Two-dimensional distributions of cationic dihedral angle and H-to-acceptor-oxygen coordination. Two-dimensional combined distribution functions of the cationic dihedral angle, $\psi_{N\text{-}C1\text{-}C2\text{-}O}$, and the donor-proton-to-acceptor-oxygen distance, $r_{H\text{-}O}$ for (a) [Ch][β-Ala], (b) [Ch][Gly], (c) [Ch][Ser], and (d) [Ch][Pro].

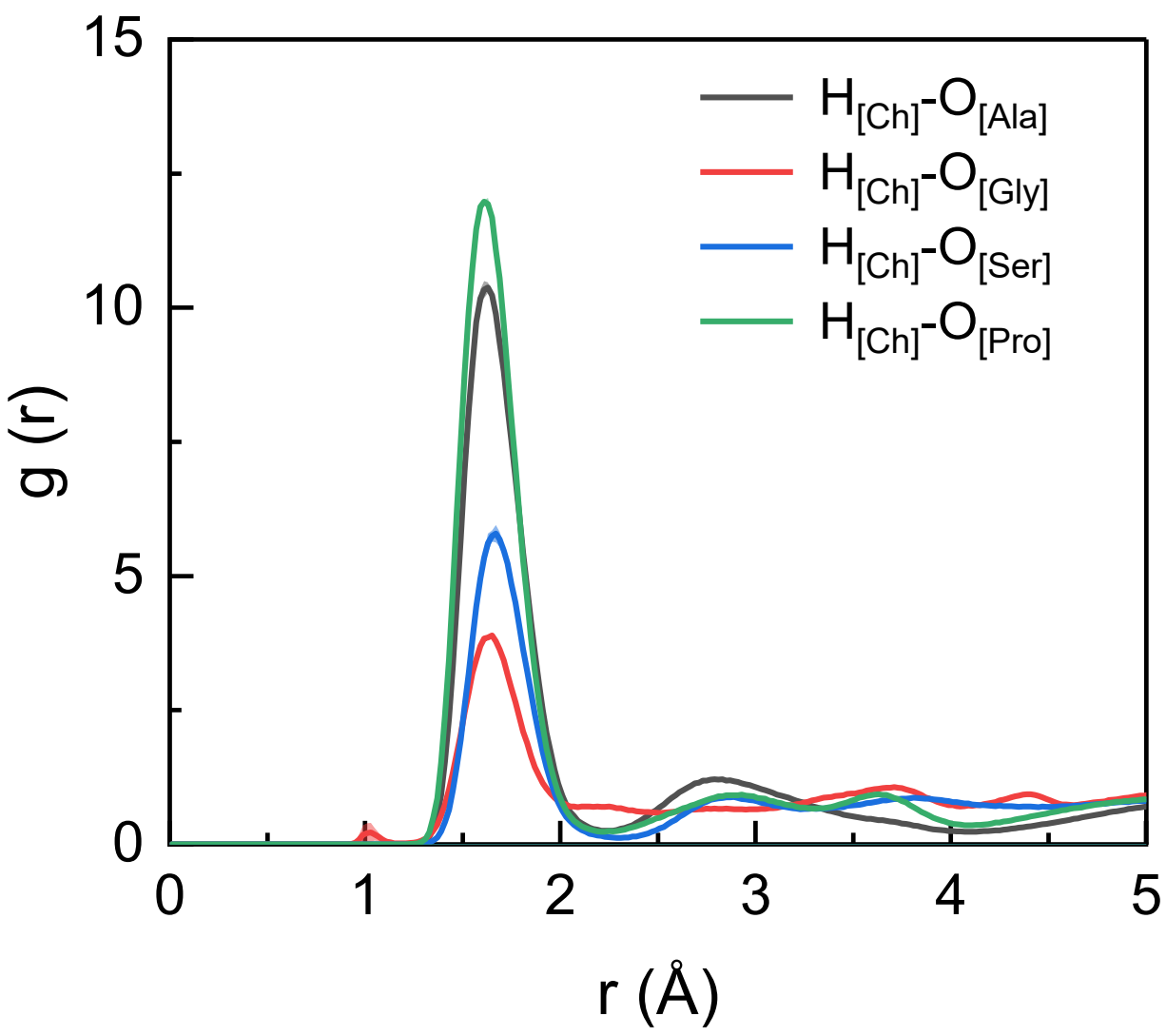

Fig. S29 RDFs between cationic protons and anionic oxygen atoms. RDFs at 303 K from NEP-MD simulations showing distances between cationic protons and anionic oxygen atoms for [Ch][*β*-Ala] (black), [Ch][Gly] (red), [Ch][Ser] (blue), and [Ch][Pro] (green).

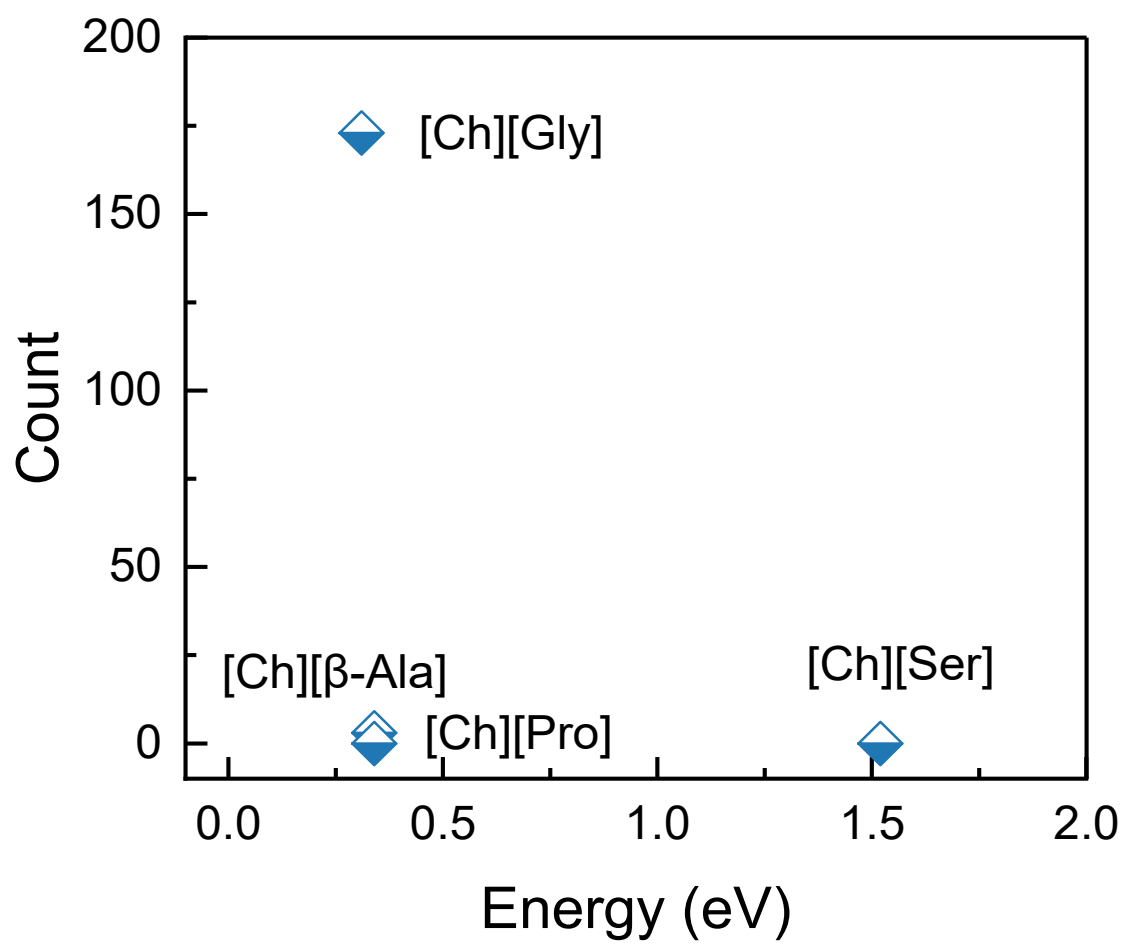

Fig. S30 Correlation between local proton-transfer frequency and free-energy barrier for the OH ↔ N pathway. Relationship between the local proton-transfer frequency and the effective free-energy barrier for the hydroxyl-to-nitrogen proton-transfer pathway.

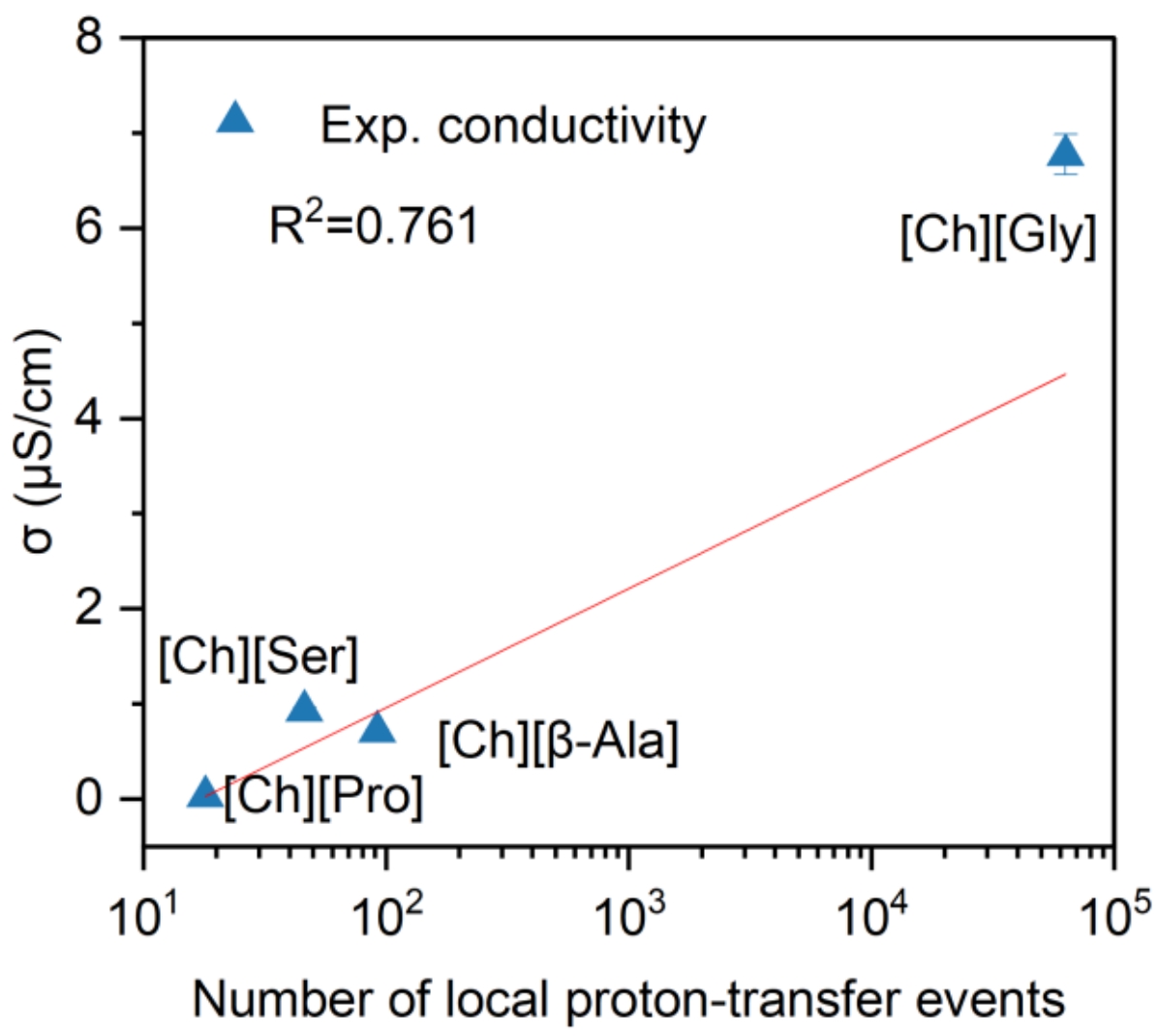

Fig. S31 Relationship between local proton-transfer activity and experimental conductivity [20]. Correlation between the number of local proton-transfer events obtained from NEP-MD simulations and the experimentally measured conductivity of the four Ch-AAILs. The positive trend suggests qualitative consistency between local proton-exchange activity and macroscopic conductivity, but local transfer counts should not be regarded as a direct quantitative measure of long-range proton transport.

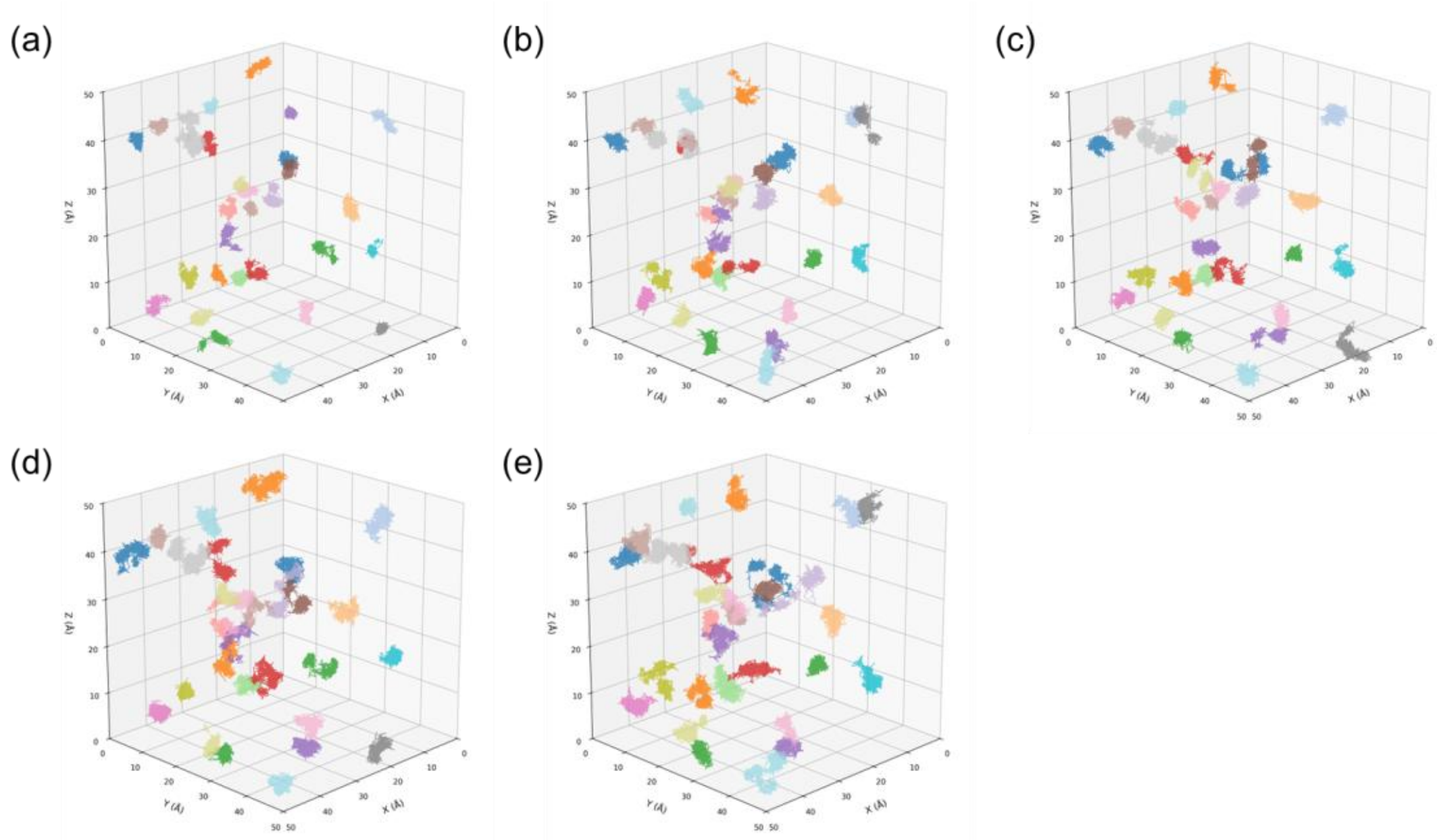

Fig. S32 Temperature-dependent spatial trajectories of selected protons in [Ch][*β*-Ala]. Spatial trajectories of ten randomly selected hydrogen atoms in [Ch][*β*-Ala] at (a) 303 K, (b) 313 K, (c) 323 K, (d) 333 K, and (e) 343 K.

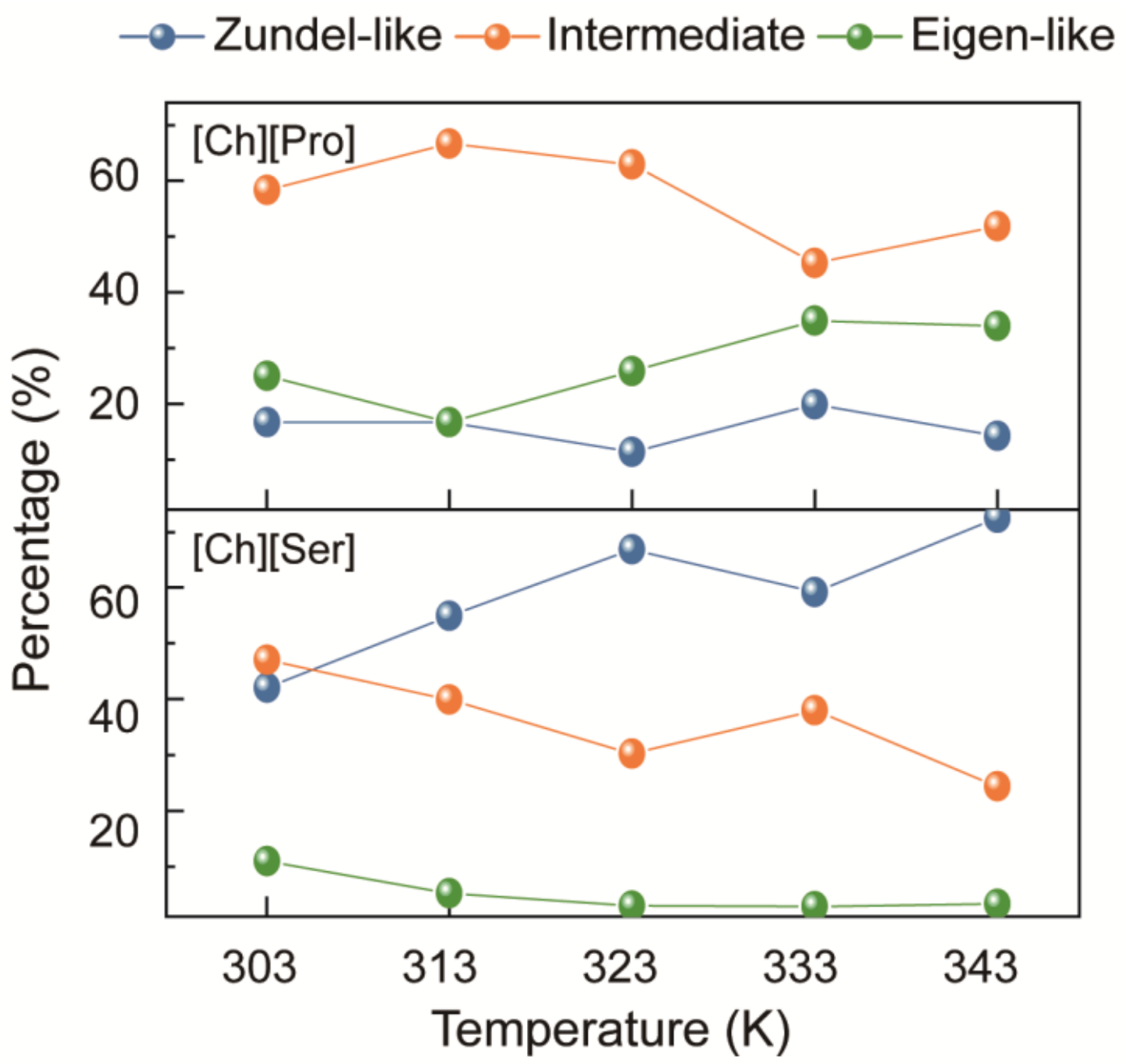

Fig. S33 Temperature-dependent populations of transient proton-solvation motifs. Populations of Zundel-like, intermediate, and Eigen-like configurations as a function of temperature for [Ch][Ser] and [Ch][Pro].

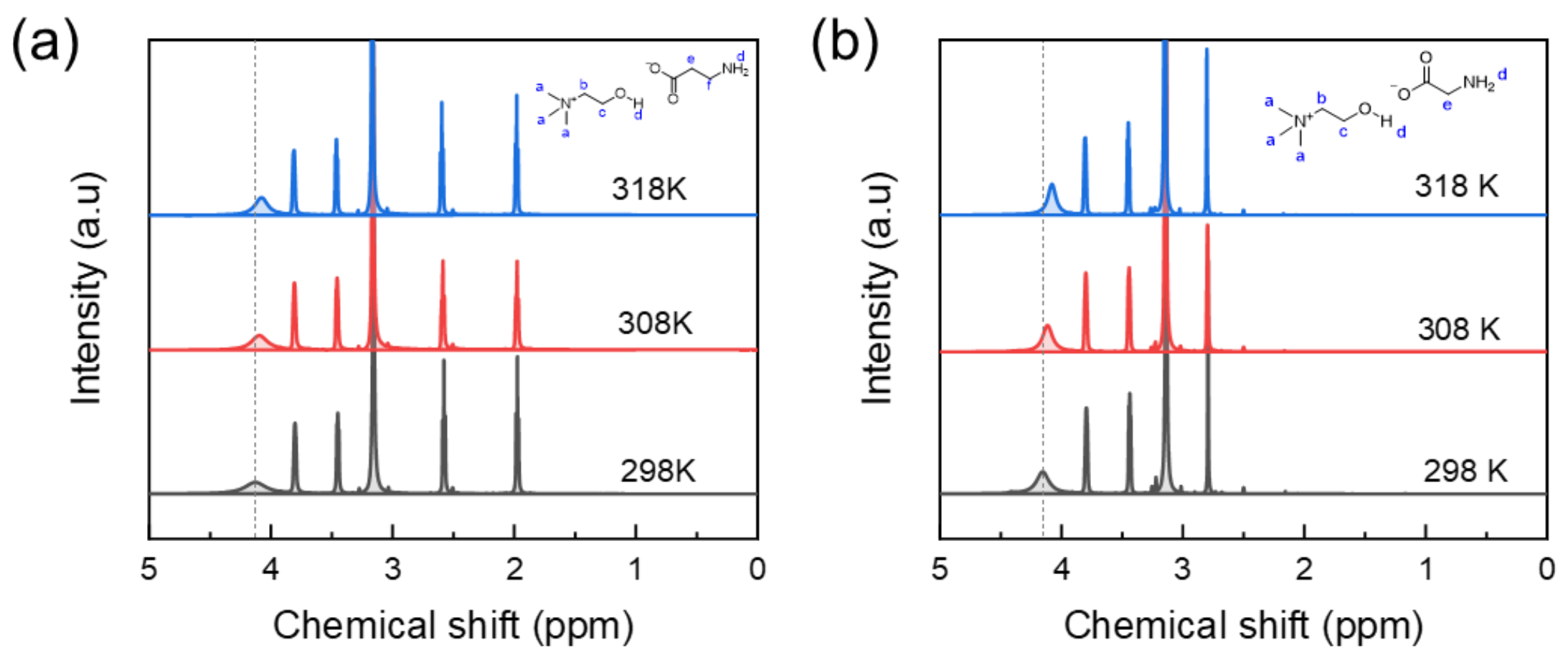

Fig. S34 Variable-temperature ^{1}H NMR spectra of [Ch][*β*-Ala] and [Ch][Gly]. Variable-temperature ^{1}H NMR spectra in the 0–5 ppm region for (a) [Ch][*β*-Ala] and (b) [Ch][Gly], recorded in DMSO-d_6 over the 3.9-4.4 ppm region at 298, 308, and 318 K, shown from bottom to top. Spectra are normalized and vertically offset for clarity. Resonance assignments for non-exchangeable protons (a–c) are indicated on the corresponding molecular structures. The exchangeable-proton resonance, peak d, highlighted by the dashed vertical line, shows a clear temperature-dependent shift, whereas the remaining resonances remain nearly unchanged.

Table S1. Hyperparameters of the NEP potentials for various PILs. Default values are used for other parameters.

ILs	r_c^R(A)	r_c^A(A)	N_{bas}^R	N_{bas}^A	n_{max}^R	n_{max}^A	$l_{3b}^{max} - l_{4b}^{max} - l_{5b}^{max}$	N_{neu}	λ_e	λ_f	N_{gen}
[Ch][*β*-Ala]	6	4	8	8	6	6	4-2-1	30	1.0	1.0	3×10^5
[Ch][Gly]	6	4	8	8	6	6	4-2-1	30	1.0	1.0	3×10^5
[Ch][Ser]	6	4	8	8	6	6	4-2-1	30	1.0	1.0	3×10^5
[Ch][Pro]	6	4	8	8	6	6	4-2-1	30	1.0	1.0	3×10^5

Table S2. GMM-derived threshold values used for the two-dimensional classification of transient proton-solvation motifs. Values are reported as mean ± standard error from three independent trajectories. δ^- denotes the proton-sharing asymmetry coordinate, $\delta^- = |R_{O_1H} - R_{O_2H}|$, and δ^+ denotes the average proton–heavy-atom distance, $\delta^+ = (R_{O_1H} + R_{O_2H})/2$. δ^-_1 and δ^+_1 define the Zundel-like/intermediate boundaries, whereas δ^-_2 and δ^+_2 define the intermediate/Eigen-like boundaries. Larger uncertainties mainly arise from limited proton-transfer statistics and trajectory-to-trajectory fluctuations in the local hydrogen-bond environment.

System	T / K	δ^-_1 / Å	δ^-_2 / Å	δ^+_1 / Å	δ^+_2 / Å
[Ch][*β*-Ala]	303	0.533 ± 0.024	0.860 ± 0.126	1.315 ± 0.012	1.523 ± 0.049
[Ch][*β*-Ala]	313	0.577 ± 0.052	0.917 ± 0.201	1.318 ± 0.003	1.510 ± 0.044
[Ch][*β*-Ala]	323	0.675 ± 0.104	1.180 ± 0.258	1.337 ± 0.013	1.608 ± 0.096

[Ch][β-Ala]	333	0.513 ± 0.035	0.745 ± 0.045	1.308 ± 0.016	1.433 ± 0.051
[Ch][β-Ala]	343	0.570 ± 0.013	1.092 ± 0.147	1.373 ± 0.048	1.680 ± 0.111
[Ch][Gly]	303	0.428 ± 0.003	0.602 ± 0.002	1.263 ± 0.002	1.332 ± 0.003
[Ch][Gly]	313	0.437 ± 0.002	0.612 ± 0.002	1.265 ± 0.000	1.335 ± 0.000
[Ch][Gly]	323	0.445 ± 0.003	0.618 ± 0.003	1.270 ± 0.000	1.335 ± 0.000
[Ch][Gly]	333	0.455 ± 0.000	0.630 ± 0.000	1.275 ± 0.000	1.350 ± 0.000
[Ch][Gly]	343	0.468 ± 0.002	0.652 ± 0.003	1.280 ± 0.000	1.375 ± 0.000
[Ch][Ser]	303	0.522 ± 0.054	0.922 ± 0.257	1.295 ± 0.026	1.463 ± 0.106
[Ch][Ser]	313	0.602 ± 0.112	1.097 ± 0.276	1.327 ± 0.042	1.555 ± 0.117
[Ch][Ser]	323	0.610 ± 0.010	1.557 ± 0.208	1.340 ± 0.005	1.785 ± 0.107
[Ch][Ser]	333	0.578 ± 0.046	1.453 ± 0.074	1.340 ± 0.013	1.762 ± 0.041
[Ch][Ser]	343	0.637 ± 0.035	1.363 ± 0.137	1.395 ± 0.041	1.792 ± 0.065
[Ch][Pro]	303	0.413 ± 0.013	0.657 ± 0.073	1.240 ± 0.005	1.330 ± 0.025
[Ch][Pro]	313	0.425 ± 0.000	0.655 ± 0.000	1.250 ± 0.000	1.340 ± 0.000
[Ch][Pro]	323	0.413 ± 0.065	0.677 ± 0.129	1.243 ± 0.009	1.337 ± 0.029
[Ch][Pro]	333	0.477 ± 0.044	0.723 ± 0.072	1.263 ± 0.025	1.350 ± 0.032
[Ch][Pro]	343	0.370 ± 0.068	0.592 ± 0.100	1.250 ± 0.019	1.337 ± 0.033

Supporting References

1. Brorsson J, Hashemi A, Fan Z *et al.* Efficient calculation of the lattice thermal conductivity by atomistic simulations with ab initio accuracy. *Adv Theor Simul*. 2022; **5**: 2100217.
2. Fan Z, Wang Y, Ying P *et al.* GPUMD: A package for constructing accurate machine-learned potentials and performing highly efficient atomistic simulations. *J Chem Phys*. 2022; **157**: 114801.
3. Fan Z. Improving the accuracy of the neuroevolution machine learning potential for multi-component systems. *J Phys: Condens Matter*. 2022; **34**: 125902.
4. Song K, Zhao R, Liu J *et al.* General-purpose machine-learned potential for 16 elemental metals and their alloys. *Nat Commun*. 2024; **15**: 10208.
5. Kresse G, Furthmüller J. Efficiency of ab-initio total energy calculations for metals and semiconductors using a plane-wave basis set. *Comput Mater Sci*. 1996; **6**: 15-50.
6. Kresse G, Furthmüller J. Efficient iterative schemes for ab initio total-energy calculations using a plane-wave basis set. *Phys Rev B*. 1996; **54**: 11169.
7. Chen C, Li Y, Zhao R *et al.* NepTrain and NepTrainKit: Automated active learning and visualization toolkit for neuroevolution potentials. *Comput Phys Commun*. 2025; **317**: 109859.
8. Plimpton S. Fast parallel algorithms for short-range molecular dynamics. *J Comput Phys*. 1995; **117**: 1-19.
9. Giełdoń A, Bobrowski M, Bielicka-Giełdoń A *et al.* Theoretical calculation of the physico-chemical properties of 1-butyl-4-methylpyridinium based ionic liquids. *J Mol Liq*. 2017; **225**: 467-474.
10. Zhou J, Liu X, Zhang S *et al.* Effect of small amount of water on the dynamics properties and microstructures of ionic liquids. *AlChE J*. 2017; **63**: 2248-2256.
11. Blöchl PE. Projector augmented-wave method. *Phys Rev B*. 1994; **50**: 17953-17979.
12. Perdew JP, Burke K, Ernzerhof M. Generalized gradient approximation made simple. *Phys Rev Lett*. 1996; **77**: 3865-3868.
13. Grimme S, Antony J, Ehrlich S *et al.* A consistent and accurate ab initio parametrization of density functional dispersion correction (DFT-D) for the 94 elements H-Pu. *J Chem Phys*. 2010; **132**: 154104.
14. Schaul T, Glasmachers T, Schmidhuber J. High dimensions and heavy tails for natural evolution strategies. In: *Proceedings of the 13th annual conference on Genetic and evolutionary computation* 2011. Dublin, Ireland. pp. 845–52.
15. Ziegler JF, Biersack JP. The stopping and range of ions in matter. In: Bromley DA (ed.) *Treatise on Heavy-Ion Science: Volume 6: Astrophysics, Chemistry, and Condensed Matter*. Boston, MA: Springer US; 1985. 93-129.
16. Grimme S, Ehrlich S, Goerigk L. Effect of the damping function in dispersion corrected density functional theory. *J Comput Chem*. 2011; **32**: 1456-1465.
17. Bernetti M, Bussi G. Pressure control using stochastic cell rescaling. *J Chem Phys*. 2020; **153**: 114107.
18. Ceriotti M, Parrinello M, Markland TE *et al.* Efficient stochastic thermostatting of path integral molecular dynamics. *J Chem Phys*. 2010; **133**: 124104.
19. Tribello GA, Bonomi M, Branduardi D *et al.* PLUMED 2: New feathers for an old bird. *Comput Phys Commun*. 2014; **185**: 604-613.

20. Tao D-J, Cheng Z, Chen F-F *et al.* Synthesis and thermophysical properties of biocompatible cholinium-based amino acid ionic liquids. *J Chem Eng Data*. 2013; **58**: 1542-1548.
